# Compromised junctional integrity phenocopies age-dependent renal dysfunction in *Drosophila Snakeskin* mutants

**DOI:** 10.1242/jcs.261118

**Published:** 2023-10-05

**Authors:** Anthony J. Dornan, Kenneth V. Halberg, Liesa-Kristin Beuter, Shireen-Anne Davies, Julian A. T. Dow

**Affiliations:** ^1^ School of Molecular Biosciences, College of Medical, Veterinary and Life Sciences, University of Glasgow, Glasgow G12 8QQ, UK; ^2^ Section for Cell and Neurobiology, Department of Biology, University of Copenhagen, Universitetsparken 15, Copenhagen DK-2100, Denmark; ^3^ Department of Animal Ecology and Systematics, Justus-Liebig-University Giessen, Giessen D-35392, Germany

**Keywords:** *Drosophila*, Malpighian tubule, Smooth septate junction, Snakeskin, Polarity, Epithelial barrier function, Ageing

## Abstract

Transporting epithelia provide a protective barrier against pathogenic insults while allowing the controlled exchange of ions, solutes and water with the external environment. In invertebrates, these functions depend on formation and maintenance of ‘tight’ septate junctions (SJs). However, the mechanism by which SJs affect transport competence and tissue homeostasis, and how these are modulated by ageing, remain incompletely understood. Here, we demonstrate that the *Drosophila* renal (Malpighian) tubules undergo an age-dependent decline in secretory capacity, which correlates with mislocalisation of SJ proteins and progressive degeneration in cellular morphology and tissue homeostasis. Acute loss of the SJ protein Snakeskin in adult tubules induced progressive changes in cellular and tissue architecture, including altered expression and localisation of junctional proteins with concomitant loss of cell polarity and barrier integrity, demonstrating that compromised junctional integrity is sufficient to replicate these ageing-related phenotypes. Taken together, our work demonstrates a crucial link between epithelial barrier integrity, tubule transport competence, renal homeostasis and organismal viability, as well as providing novel insights into the mechanisms underpinning ageing and renal disease.

## Teaser

**Summary:** In *Drosophila*, age-related decreased kidney function is due to loss of epithelial barrier integrity and failure of fluid homeostasis, effects phenocopied by cell-specific knockdown of the essential junctional protein Snakeskin.

## INTRODUCTION

In multicellular animals, ageing presents as a progressive decline in tissue homeostasis and organ function, leading to increasing probability of disease and death (Rera et al., 2013b; Rose et al., 2012). Regulation of tissue homeostasis is thus critical to organismal lifespan, yet the molecular and cellular activities responsible for mediating age-dependent changes in tissue function, and how this in turn impacts organismal longevity, remain largely unexplored.

Maintenance of a healthy intestine has recently emerged as a critical determinant of lifespan across taxa, with stereotypic hallmarks of intestinal ageing including augmented stem cell behaviour, blocked terminal differentiation, activation of inflammatory pathways, reduced nutrient uptake, loss of barrier integrity and dysbiosis (Clark and Walker, 2018; Clark et al., 2015; Davies et al., 2012; Hu and Jasper, 2017; McGee et al., 2011; Regan et al., 2016; Rera et al., 2013a, 2012; Resnik-Docampo et al., 2017, 2018; Salazar et al., 2018). Notably, a causal link between these phenotypes and age-related remodelling of cell-to-cell junctions has been established in proliferative tissues such as the intestine (Clark et al., 2015; Izumi et al., 2019; Resnik-Docampo et al., 2017, 2018; Salazar et al., 2018), indicating that dysregulation of junctional proteins in self-renewing tissues might be a principal driver of ageing. However, whether age-related changes in cell-to-cell contacts also occur in tissues defined by low or no cell turnover, such as the nervous system, heart and kidneys, and whether such alterations significantly contribute to tissue degeneration and/or ageing remains unresolved.

In insects, the renal (Malpighian) tubules (MTs) constitute the functional analogue of the vertebrate kidneys (Cohen et al., 2020; Dow et al., 2018) and are, as in vertebrates, considered to be a non-proliferative tissue (Skaer, 1993). The MTs are the principal organs responsible for maintaining water and ion homeostasis, yet serve additional roles in xenobiotic detoxification (Terhzaz et al., 2015; Yang et al., 2007) and immunity (Verma and Tapadia, 2014). In the fruit fly *Drosophila melanogaster*, the MTs consist mainly of two physiologically distinct secretory cell types: the principal cell (PC) and the intercalated or ‘stellate’ cell (SC) (Beyenbach et al., 2010; Cohen et al., 2020; Dow, 2012; Sözen et al., 1997). PCs are the sites of active cation transport, energised by V-ATPases localised apically to a prominent brush border (Cohen et al., 2020; Halberg et al., 2016; Naseem et al., 2022), whereas the smaller SCs control channel-mediated Cl^−^ and water fluxes (Beyenbach et al., 2010; Cabrero et al., 2020; Cohen et al., 2020; Dow, 2012). Primary urine production in *Drosophila* is achieved through the integrated actions of PCs and SCs, driving transepithelial transport of ions and water. A third population of ‘tiny’ cells, found only in the ureter and lower tubule, have been proposed to be renal stem cells (Cohen et al., 2020; Martínez-Corrales et al., 2019; Singh and Hou, 2009, 2008; Singh et al., 2011; Takashima et al., 2013; Wang and Spradling, 2020), yet to what extent these potentially proliferative cells contribute to tissue repair and/or homeostasis is unclear.

In invertebrates, epithelial integrity is maintained by either pleated (p) or smooth (s) septate junctions (SJs), lateral intercellular contacts that are functional analogues of vertebrate tight junctions (Furuse and Izumi, 2017), with sSJs being the dominant junctional complex in MTs (Beyenbach et al., 2020; Jonusaite et al., 2020; Lane and Skaer, 2016; Noirot-Timothee and Noirot, 1980). The occluding junction protein Snakeskin (Ssk) along with its counterpart Tetraspanin 2a (Tsp2a) and the cell adhesion protein Mesh are critical for the proper formation of sSJs (Beyenbach et al., 2020; Izumi et al., 2019, 2016, 2012; Jonusaite et al., 2020; Salazar et al., 2018; Xu et al., 2019; Yanagihashi et al., 2012). Mesh, Ssk and Tsp2a have been shown, in ectodermally derived pSJs, to be necessary, both individually and as a complex, for the appropriate localisation of associated junctional proteins such as Lethal giant larvae [Lgl or L(2)gl] and Discs large (Dlg or Dlg1), both part of the Scribble polarity module (Khoury and Bilder, 2020; Oshima and Fehon, 2011), Coracle (Cora) and Fasciclin III (FasIII or Fas3) (Chen et al., 2018; Izumi et al., 2016, 2012; Salazar et al., 2018; Yanagihashi et al., 2012). Recently, an additional component of the Ssk–Mesh–Tsp2a complex necessary for appropriate formation of sSJs, Hoka (CG13704), has been identified in the midgut and MTs (Izumi et al., 2021). However, it is thought that, rather than being a core component indispensable for sSJ formation, Hoka might function to facilitate appropriate apicolateral positioning of the Ssk–Mesh–Tsp2a complex (Izumi et al., 2021).

Here, we describe ageing in the insect renal tubule and demonstrate that adult MTs undergo an age-dependent decline in secretory transport capacity, which correlates with mislocalisation of SJ proteins and coincident progressive degeneration in cellular morphology and overall tissue homeostasis. As the ageing phenotype was reminiscent of that observed in junctional mutants, we further showed that these effects were phenocopied by acute loss of *Ssk* expression in either PC or SC sub-populations of adult MTs. *Ssk* impairment resulted in mislocalisation of junctional components, again manifesting in overt degeneration in cellular and tissue morphology. Critically, this acute failure of junctional integrity led to an accelerated reduction in secretory capacity and concomitant loss of systemic fluid homeostasis, which ultimately resulted in a significant reduction in organismal lifespan. Furthermore, cell-specific manipulations of *Ssk* expression led to a pronounced increase in SC clustering, a block in SC maturation and a loss in apicobasal polarity, indicating a key role for *Ssk* in maintaining MT function and stability. Finally, knocking down *Ssk* expression also led to a striking proliferation of tiny (renal stem) cells as well as a dramatic increase in tubule tracheation, suggesting that MTs can autonomously respond to tissue damage and that Ssk acts as a novel regulator of tissue homeostasis in the tubule. Taken together, our work demonstrates a crucial link between cell–cell junction integrity, epithelial transport competence and tubule homeostasis in a classically non-proliferative tissue, which provides novel insights into the mechanisms underlying tissue degeneration and ageing.

## RESULTS

### Cell-specific Ssk depletion impairs systemic osmoregulation and reduces organismal lifespan

**Fig. 1.**
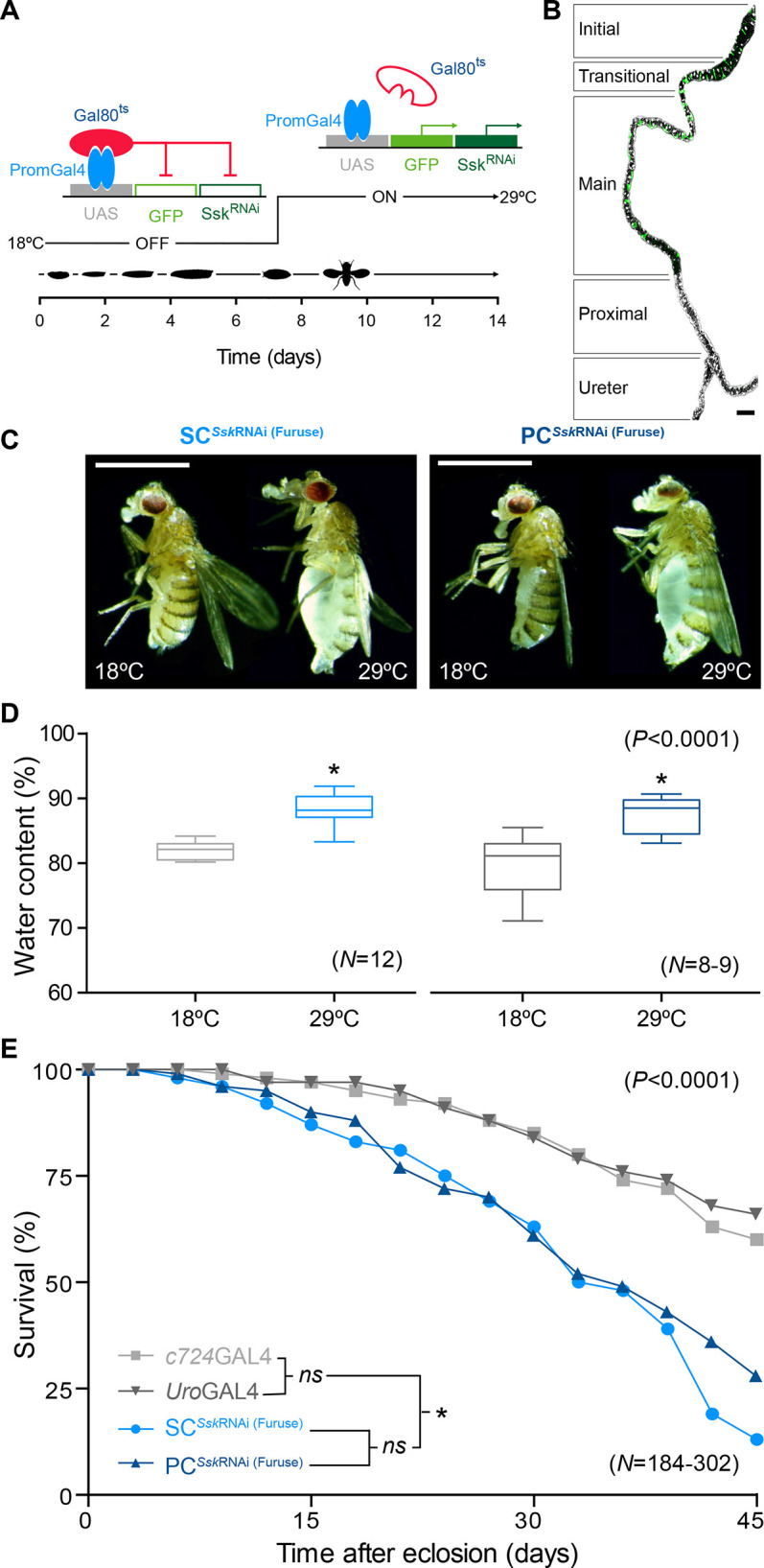
**Cell-specific *Ssk* depletion in adult MTs results in loss of fluid integrity and significantly reduced viability.** (A) Temperature-sensitive stratagem for gene silencing of *Snakeskin* using *c724*GAL4 and *Uro*GAL4 drivers specifically in stellate cells (SCs) or principal cells (PCs) of adult MTs, respectively. GAL4 (PromGAL4) driver expression is repressed at 18°C (permissive temperature) by expression of *tubPGAL80^ts^*, precluding expression of GAL4-responsive UAS-transgenes (*UAS-mCD8::GFP* and *UAS-Ssk*^RNAi^; red line). Experimental animals were transferred at late L3 or white pre-pupal stage to 29°C (restrictive temperature), repressing *tubPGAL80^ts^* expression, allowing expression of the GAL4-responsive UAS transgenes in a spatially restricted manner (green arrows). (B) *c724*GAL4 driving membrane-bound GFP (mGFP) expression in a 5-day-old adult MT. Maximal *z*-projection of one half of an anterior tubule pair (with the other tubule cropped out) with the ureter included is shown. The functional domains are delineated. Note the bar-shaped cells in the initial and transitional segment, SCs in the transitional and main segment, and absent mGFP (green) expression in the proximal segment and the ureter. DAPI staining all cell nuclei is shown (white). The image is representative of 25 animals. Scale bar: 100 µm. (C) SC*^Ssk^*^RNAi^ and PC*^Ssk^*^RNAi^ experimental adult females (29°C) exhibited an overt bloated abdomen phenotype compared to controls (18°C). Scale bars: 2 mm. (D) Comparison of ‘wet’ to ‘dry’ weight differences for SC*^Ssk^*^RNAi^ and PC*^Ssk^*^RNAi^ experimental adult females (29°C) versus controls (18°C). This bloating phenotype was reiterated using the *Ssk*^RNAi (GD)^ and *Ssk*^RNAi (KK)^ transgenic lines (Fig. S2C). Boxes show the 25–75th percentiles, whiskers show the minimum and maximum values, and the median is marked with a line. Each individual set consisted of 20 flies. (E) Survival assay demonstrating significant reduction in viability of SC*^Ssk^*^RNAi^ and PC*^Ssk^*^RNAi^ experimental adult females versus that of controls. Note that haemolymph accumulation resulting in a bloated abdomen occurred in both males and females. *n-*values are shown in parentheses. ns, not significant; *P<0.0001 [two-tailed unpaired Student's *t*-test for D; Mantel–Cox (log-rank) test for E].

Failure to form sSJs properly in early development, either ubiquitously or restricted to the MTs, is lethal (Beyenbach et al., 2020; Jonusaite et al., 2020; Izumi et al., 2019; Yanagihashi et al., 2012). We therefore employed temperature-sensitive tubulin GAL80 (McGuire et al., 2004), in conjunction with either an SC-specific (*c724*GAL4) (Sözen et al., 1997) or PC-specific (*Uro*GAL4) (Terhzaz et al., 2010) GAL4 driver acting on three independent UAS-*Ssk* RNAi constructs, *Ssk*^RNAi (GD)^, *Ssk*^RNAi (KK)^ (Dietzl et al., 2007) and *Ssk*^RNAi (Furuse)^ (Yanagihashi et al., 2012), to restrictively knock down *Ssk* expression in only adult tubules (Fig. 1A). At the permissive temperature (18°C), GAL4 expression was repressed and *c724*GAL4>GAL80^ts^>*Ssk*^RNAi^ and *Uro*GAL4>GAL80^ts^>*Ssk*^RNAi^ (respectively designated as SC*^Ssk^*^RNAi^ and PC*^Ssk^*^RNAi^) flies developed into viable, fertile adults (Fig. 1; Fig. S1). However, when transferred to the restrictive temperature (29°C), at the pre-pupal stage, GAL4 drove expression of *Ssk*^RNAi^ in a tissue- and developmentally restricted manner (Fig. 1; Fig. S1). This intersectional stratagem was designed to nullify any off-target effects associated with expression of the individual GAL4 drivers and UAS reporters in other tissues, ensuring that the phenotypes observed were specific to the cells and tissues of the tubule (Fig. 1B). The three drivers used show highly enriched, if not absolute, expression in the tubules (FlyAtlas 2; Leader et al., 2018). We used the temperature-sensitive GAL80 construct to ensure that GAL4 expression only occurred from the pupa onwards, avoiding earlier developmental effects, and driving expression of three different RNAi constructs directed at *Ssk*, the expression of which is again highly enriched in the gut and MTs (FlyAtlas 2; Leader et al., 2018). These variant GAL4 drivers, knocking down expression of the smooth SJ component Snakeskin in the adult fly, resulted in a common set of phenotypes, complementing those described to occur during intestinal barrier dysfunction, that affected tubule physiology to impact organismal viability. These SC*^Ssk^*^RNAi^ and PC*^Ssk^*^RNAi^ experimental animals successfully eclosed as adults. However, over time, they progressively developed a ‘bloated’ phenotype due to increased water content (Fig. 1C,D; Fig. S2C), a clear indicator of compromised MT epithelial function, which resulted in significantly reduced viability (Fig. 1E).

### Cell-specific Ssk depletion compromises cellular morphology

**Fig. 2.**
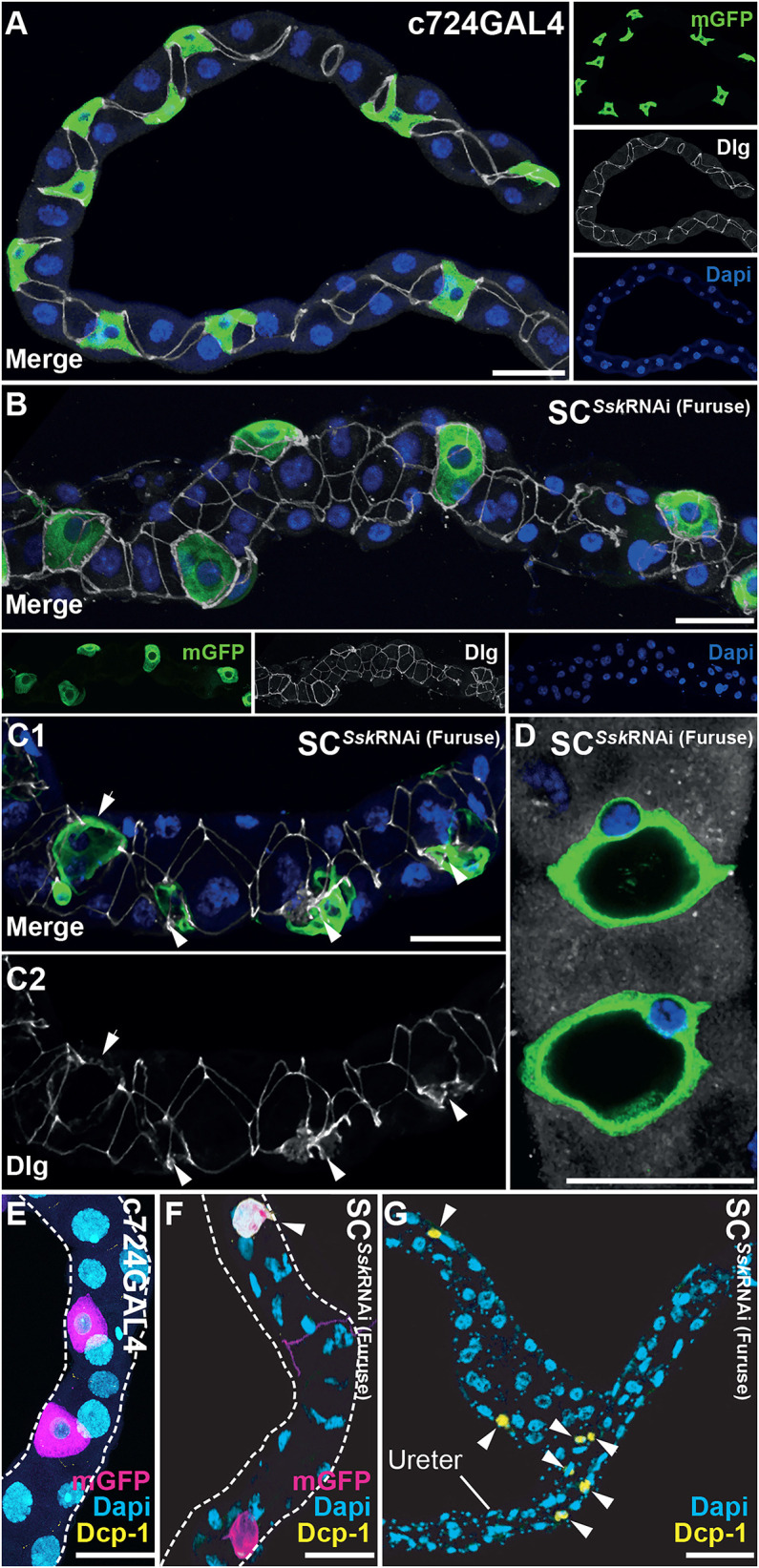
**SC-specific *Ssk* depletion in adult MTs results in compromised cellular morphology.** (A) *c724*GAL4 driving mGFP expression in 5-day-old adult MTs, with expression in evenly spaced SCs exhibiting stereotypical stellar morphology and smoothly organised junctions throughout, as indicated by anti-Discs large (Dlg) staining. (B) A representative 5-day-old adult SC*^Ssk^*^RNAi^ MT. SCs exhibited absence of mature stellar morphology and were extruded from the plane of the MT. Junctional complexes appeared disorganised or missing. (C1,C2) A representative 5-day-old adult SC*^Ssk^*^RNAi^ MT highlighting accretions of Dlg associated with mutant SCs (C2; arrowheads). (D) A subset of a *z*-stack of a SC*^Ssk^*^RNAi^ MT detailing ‘inflated’ vacuolar mutant SCs. For A–D, mGFP, green; Dlg, white; DAPI, blue. (E) Detail of the main segment with *c724*GAL4 driving mGFP expression in a 5-day-old adult MT, demonstrating lack of expression of the apoptotic marker Death caspase-1 (Dcp-1). Note that this absence of Dcp-1 expression was also apparent in the associated ureter and proximal (lower) segment of control tubules. (F,G) Details of main segment (F) and the ureter and proximal segment (G) of 5-day-old adult SC*^Ssk^*^RNAi^ MTs, demonstrating expression of Dcp-1 in an SC and RNSCs (arrowheads). For E–G, mGFP, magenta; Dcp-1, yellow; DAPI, blue. Images in A–G are representative of 25 animals. All scale bars: 50 µm.

We then set out to determine the specific cellular deficits that occurred when Ssk was depleted in adult MTs that might contribute to this compromised epithelial function. When *Ssk* was knocked down in either SCs or PCs of adult tubules, junctional complex organisation – as realised by staining for Dlg, a junctional protein required for structure, cell polarity and proliferation control in epithelia (Bilder et al., 2000; Khoury and Bilder, 2020; Woods et al., 1996) – was overtly compromised compared to the regular distribution exhibited in controls (Figs 1B and 2A–C; Figs S2A,B and S3), with mislocalisation of Dlg apparent as accretions in the cytoplasm (Fig. 2B,C; Figs S2A,B and S3B,C). Notably, SCs from SC*^Ssk^*^RNAi^ animals failed to develop their stereotypic mature ‘stellar’ morphology, instead appearing cuboidal and extruded from the tubule (Fig. 2B,C; Fig. S2A,B). This extrusion might be the consequence of inflation of an internal vacuole, which dramatically increased cellular volume (Fig. 2D).

Notably, these results were also observed using a second SC-specific GAL4 driver, ClC-aGAL4 (Cabrero et al., 2020), which reiterated the phenotypic profile associated with SC-specific knockdown of Ssk, albeit to a lesser extent, likely due to ClC-aGAL4 acting as a weaker driver compared with *c724*GAL4. That is, ClC-aGAL4>GAL80^ts^ >*Ssk*^RNAi^ experimental adults developed a bloated abdomen due to an accumulation of haemolymph in the abdomen, indicative of loss of tubule epithelial integrity, distended MTs with disorganised junctional complexes, and cuboidal SCs with associated loss of apicobasal polarity and the ClC-a channel (Fig. S3).

Over time, the SC*^Ssk^*^RNAi^ and PC*^Ssk^*^RNAi^ mutant cells appeared to collapse in on themselves. In conjunction with this, expression of membrane-bound GFP (mGFP; driven by *Uro*GAL4) and Dlg diminished and was finally lost, with loss of nucleation, as evidenced by loss of DAPI staining (Fig. S4B,C). In addition, we observed affected cells expressing the apoptotic marker Death caspase-1 (Dcp-1) (Song et al., 1997), indicating that these cells are undergoing apoptosis (Fig. 2E–G).

### Ssk depletion promotes failure of the junctional complex, resulting in loss of cellular cytoarchitecture and apicobasal polarity

**Fig. 3.**
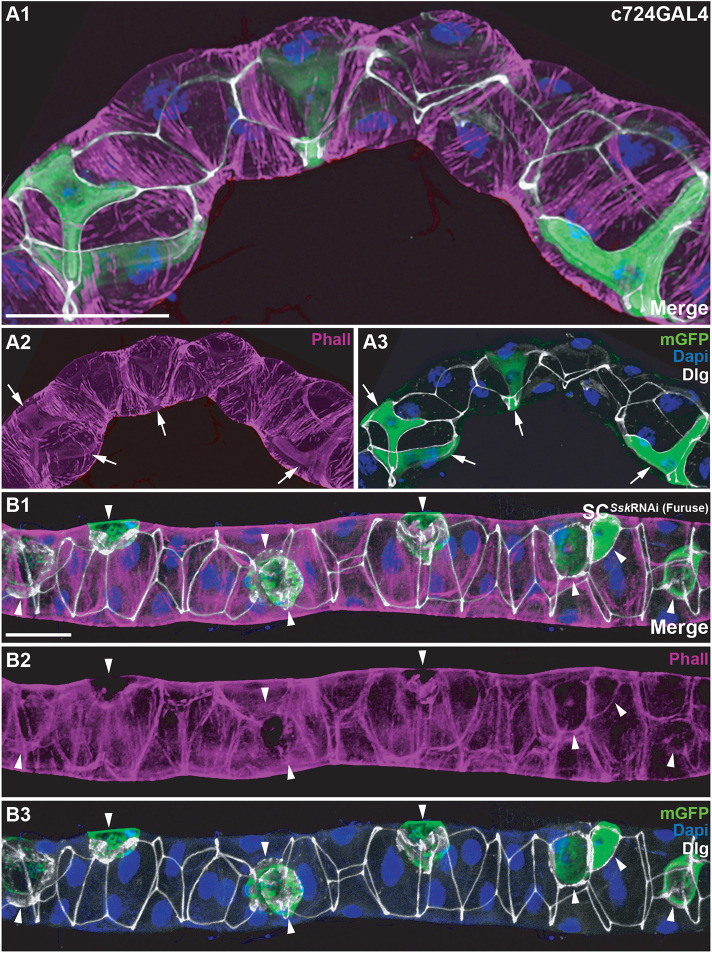
**SC-specific depletion of *Ssk* in adult MTs results in loss of cytoarchitectural organisation.** (A1–A3) *c724*GAL4 driving mGFP expression in a 5-day-old adult MT with cellular architecture strongly associated with cellular junctions, as observed by Phalloidin (Phall; F-actin) staining. A2 and A3 highlight the internal cytoarchitecture specific to SCs (arrows). (B1–B3) A 5-day-old adult SC*^Ssk^*^RNAi^ MT highlighting loss of F-actin associated with cellular junctions and SCs. B1 and B2 highlight the absence of internal SC cytoarchitecture (arrowheads). Accretions of Dlg overt in degenerating SCs with reduced GFP expression and loss of DAPI staining. mGFP, green; Phall, magenta; Dlg, white; DAPI, blue. Images in A1–B3 are representative of 25 animals. Scale bars: 50 µm.

As it has been shown that dysregulation of Dlg results in disruption of microfilament-maintained cytoarchitecture (Izumi et al., 2019; Woods et al., 1996) (Fig. 3A), we examined MTs in which *Ssk* had been knocked down and found that F-actin microfilaments were clearly absent in SC*^Ssk^*^RNAi^ SCs, whereas filaments associated with bicellular boundaries appeared reduced (Fig. 3B; Fig. S2D). This loss of F-actin microfilaments was also observed in PC*^Ssk^*^RNAi^ MTs (Fig. S4C).

**Fig. 4.**
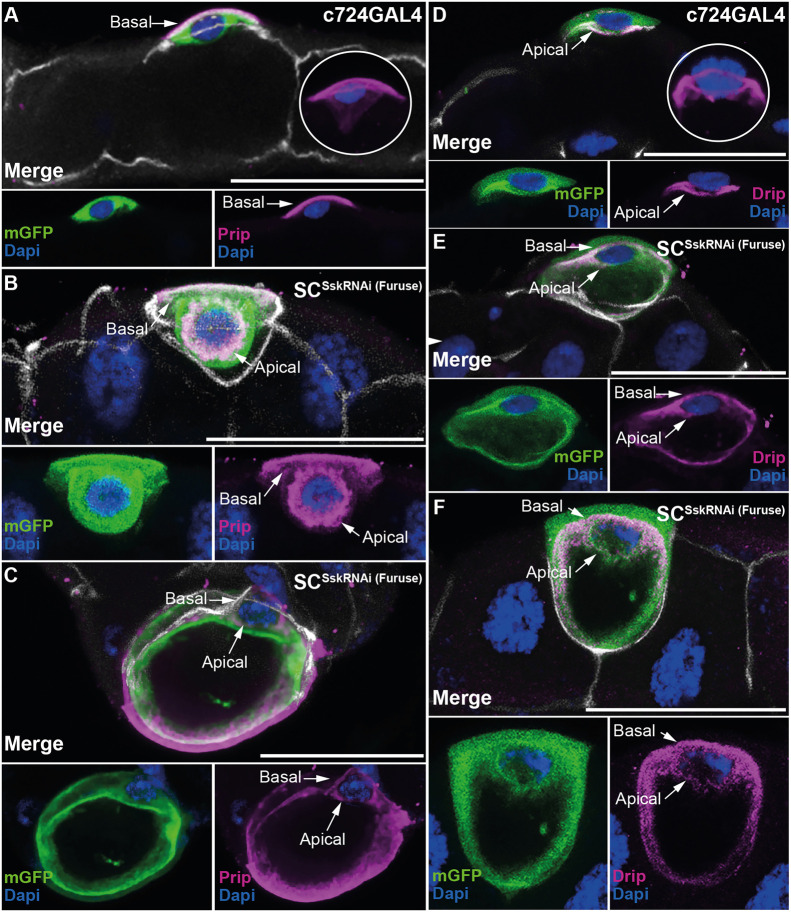
**Cell-specific depletion of *Ssk* in adult MTs results in loss of apicobasal polarity.** (A) A *z*-stack subset of a control MT from a 5-day-old *c724*GAL4 adult expressing mGFP, detailing restricted localisation of the aquaporin channel Prip to the basolateral (basal, arrow) membrane of the SC. The maximal *z*-stack (inset) demonstrates the relationship of the basolaterally located Prip channel with respect to the nucleus, as observed by DAPI staining. (B) A *z*-stack subset of a 5-day-old SC*^Ssk^*^RNAi^ adult MT, demonstrating Prip localisation to the basolateral membrane (basal, arrow) and also mislocalisation to the apical membrane (apical, arrow). (C) A *z*-stack subset of a 5-day-old SC*^Ssk^*^RNAi^ adult MT, demonstrating Prip localisation not only to the basolateral membrane (basal, arrow), but also mislocalisation apically (apical, arrow), with this mislocalisation extending to encapsulate the expanded internal vacuole. (D) A *z*-stack subset of a control MT from a 5-day-old *c724*GAL4 adult expressing mGFP, detailing restricted localisation of the aquaporin channel Drip to the apical (apical, arrow) membrane of the SC, associated with the MT lumen. The maximal *z*-stack (inset) demonstrates the relationship of the apically located Drip channel with respect to the nucleus, as observed by Dapi staining. (E) A *z*-stack subset of a 5-day-old SC*^Ssk^*^RNAi^ adult MT, demonstrating Prip localisation to the apical membrane (apical, arrow) and also mislocalisation to the basolateral membrane (basal, arrow). (F) A *z*-stack subset of a 5-day-old SC*^Ssk^*^RNAi^ adult MT, demonstrating Drip localisation not only apically (Apical, arrow), but also mislocalisation to the basolateral membrane (basal, arrow), with this mislocalisation also extending to encapsulate the expanded internal vacuole. mGFP, green; Dlg, white; Prip (A–C) and Drip (D–F), magenta; DAPI, blue. Images in A–F are representative of 25 animals. Scale bars: 50 µm.

Dysregulation of Dlg and associated loss of cytoarchitecture also results in failure of apicobasal polarity (Laprise and Tepass, 2011). To test for loss of cell polarity, we employed two antibodies directed against the aquaporin channels Prip and Drip, which are specific, respectively, to the basolateral and apical membranes of SCs (Fig. 4A,D) (Cabrero et al., 2020). Mutant SC*^Ssk^*^RNAi^ SCs showed clear mislocalisation of both Prip and Drip, with expression apparent not only at the expected cell membrane but also anomalously at the opposing membrane (Fig. 4B,C,E,F; Fig. S2E,F). This mislocalisation of the aquaporin channels was also clearly associated with the vacuole within the mutant cell (Fig. 4C,F; Fig. S2F), the enlargement of which might result from an internalised flow of misdirected water occurring via these mislocalised channels.

To further demonstrate loss of cell polarity, we used an antibody to the Na^+^/K^+^ ATPase α-subunit (antibody binds to the α-subunit of all Na^+^/K^+^ ATPase isoforms; a5, DSHB), a transporter known to localise to the basolateral membrane of insect tubules (Halberg and Mobjerg, 2012; Torrie et al., 2004; Patrick et al., 2006). We observed an overall reduction of Na^+^/K^+^ ATPase expression in SC*^Ssk^*^RNAi (Furuse)^ MTs compared to that in controls, with a marked decrease in basal localisation in SCs, again indicative of a loss of apicobasal polarity (Fig. S5). The observed loss of apical bias indicated by GFP expression in experimental PC*^Ssk^*^RNAi (Furuse)^ MTs compared with that in controls (Fig. S4A,B) is consistent with the demonstrated loss of apicobasal polarity in SC*^Ssk^*^RNAi^ MTs. This loss of cellular polarity is unsurprising, considering the functional requirement for proper formation of junctional complexes in determining overall cellular polarity (Bonello et al., 2019; Nelson, 2003).

In SCs, kinin-modulated Cl^−^ shunt conductance occurs specifically through the chloride channel ClC-a localizing to the basolateral membrane (Fig. S6A) (Cabrero et al., 2020, 2014). We attempted to use anti-ClC-a to assay polarity in SC populations in experimental adult MTs and found that ClC-a expression was completely absent in the cuboidal SC population in SC*^Ssk^*^RNAi (Furuse)^ MTs (Fig. S6B) but apparently unaffected in SCs in PC*^Ssk^*^RNAi (Furuse)^ MTs (Fig. S6C). To test the physiological significance of these cellular defects, we exposed animals to conditions known to induce osmotic stress and assayed for organismal survival. These results revealed that SC*^Ssk^*^RNAi (Furuse)^ adults demonstrated a significant reduction in survival when allowed access to water only (non-desiccating starvation) (Fig. S6D) but, intriguingly, this compromised viability was absent when the adults were exposed to high salt loading (Fig. S6E). These data are consistent with observed defects in SC function and compromised hormone-induced changes in Cl^−^ and water fluxes (Cabrero et al., 2014; Denholm et al., 2013; Feingold et al., 2019), which result in a reduced capacity to respond and adapt to hypoosmotic challenges and regulate systemic fluid balance. Taken together, our data demonstrate a necessary role for *Ssk* in maintaining the SC transport machinery and, by extension, tubule transport competency and organismal fluid balance.

### Cell-specific depletion of Ssk results in absence of septa and impairment of junctional, cellular and tissue organisation

**Fig. 5.**
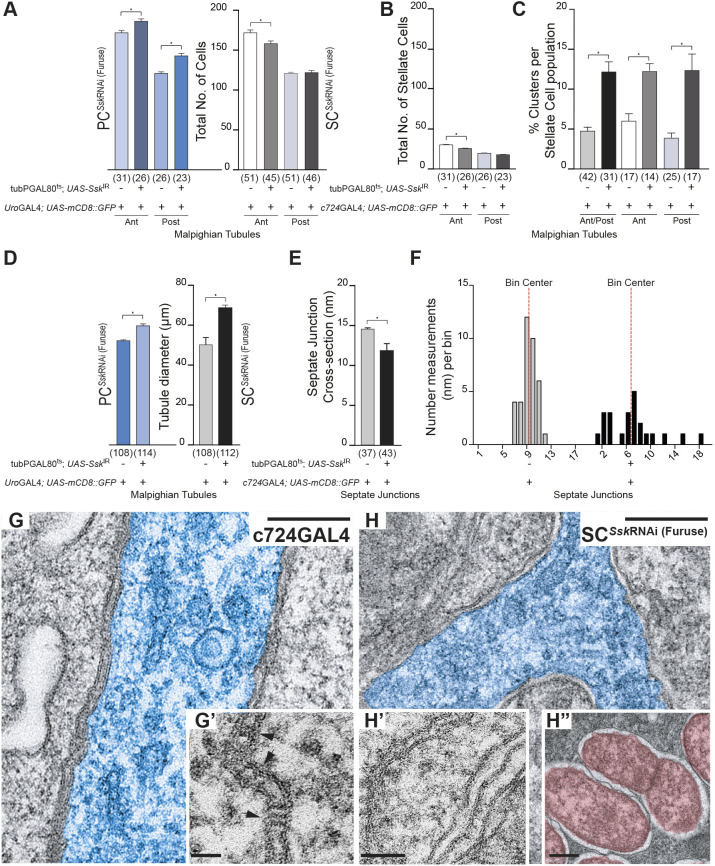
**Cell-specific depletion of *Ssk* in adult MTs results in failure of septa, junction, cell and tissue organisation.** (A) Comparison of total cell counts for SC*^Ssk^*^RNAi (Furuse)^ and PC*^Ssk^*^RNAi (Furuse)^ MTs compared to those of controls. Both anterior and posterior PC*^Ssk^*^RNAi^ MTs exhibited a significant increase in cell populations compared with those of controls (186±3 cells, *n*=26, versus 171±4 cells, *n*=31, *P<0.005 for anterior tubules; 143±4 cells, *n*=23, versus 121±2 cells, *n*=26, *P<0.005 for posterior tubules). Anterior SC*^Ssk^*^RNAi^ MTs exhibited a significant decrease in cell populations, whereas there were no changes in posterior tubules compared with controls (158±4 cells, *n*=45, versus 174±2 cells, *n*=51, *P<0.0001 for anterior tubules; 122±3 cells, *n*=46, versus 121±2 cells, *n*=51, not significant for posterior tubules). (B) Anterior SC*^Ssk^*^RNAi^ MTs exhibited a significant decrease in SC populations, whereas there were no changes in posterior tubules compared with controls (SC counts: 25±1, *n*=45, versus 30±1, *n*=51, *P<0.0002 for anterior tubules; 18±1, *n*=46, versus 19±1, *n*=51, not significant for posterior tubules). (C) Both anterior and posterior SC*^Ssk^*^RNAi^ MTs exhibited a significant increase in the percentage of SC cell clusters compared with that of controls (12.2±1.27%, *n*=31, versus 4.7±0.59%, *n*=42, *P<0.0001). A cluster was defined as two or more adjoining SCs. (D) Both anterior and posterior PC*^Ssk^*^RNAi^ and SC*^Ssk^*^RNAi^ MTs exhibited a significant increase in tubule cross-sectional diameters compared with those of controls (69.10±1.184 nm, *n*=114, versus 50.30±3.883 nm, *n*=108, *P*<0.0001, and 60.24±0.8628 nm, *n*=112, versus 52.75±0.6783 nm, *n*=108, *P*<0.0001, respectively). (E) SC*^Ssk^*^RNAi (Furuse)^ septate junctions (SJs) exhibited a significant decrease in cross-sectional distance as compared with that in controls (11.77±0.868 nm, *n*=43, versus 14.43±0.199 nm, *n*=37; *P*<0.0001). (F) Junctional spans for SC*^Ssk^*^RNAi (Furuse)^ MTs were highly irregular, as evidenced by the frequency-distribution in SJ measurements. *P<0.0001. For A–F, data show the mean±s.e.m. and *n*-values are shown in parentheses. Two-tailed unpaired Student's *t*-test was used to determine significance. (G) TEM of the main segment of a 5-day-old *c724*GAL4 control MT featuring the SJs associated with an SC (pseudocolour, blue), demonstrating the regular spacing and stereotypical ladder appearance caused by the presence of the septa. (G′) Inset: higher-magnification image highlighting the presence of septa (arrows) spanning the SJs. (H) TEM of the main segment of a 5-day-old SC*^Ssk^*^RNAi (furuse)^ MT featuring the septal junctions associated with a SC (pseudocolour, blue), demonstrating disorganised spacing and absence of septa ladders. (H′) Inset, higher-magnification image highlighting the absence of septa associated with mutant SJs. (H″) Inset: higher-magnification image highlighting the presence of a dividing bacterium (psuedocolour, red). Images in G–H″ are representative of 10 animals. Scale bars: 0.2 µm (G,H); 50 nm (G′,H′,H″).

We next set out to determine the cellular mechanisms through which knockdown of *Ssk* in specific sub-population of cells in the adult MTs could affect overall tissue morphology and homeostatic capabilities. Although both SC*^Ssk^*^RNAi^ and PC*^Ssk^*^RNAi^ MTs appeared hyperplastic (Fig. 2B; Fig. S4B), there was only a small, though significant, increase in the overall population of cells in PC*^Ssk^*^RNAi^ MTs (Fig. 5A). In contrast, there was a slight decrease in the SC population in anterior SC*^Ssk^*^RNA^ MTs (Fig. 5A,B), presumably due to progressive loss of SCs through apoptosis.

In SC*^Ssk^*^RNAi^ SCs, not only was mature stellar morphology affected, but so too was spatial distribution, with a significant increase in the percentage of the SC population exhibiting clustering of two or more cells (Fig. 5C). Tubules are traditionally regarded as developmentally ‘static’, that is, the SC population has intercalated and been positioned within the tubule primordia by mid-embryogenesis and SCs are thought merely to ‘mature’ physiologically (developing their characteristic stellar or bar morphology during the final stages of pupariation) (Cohen et al., 2020; Denholm, 2013; Denholm et al., 2013, 2003; Dow, 2012). This dysregulation in positioning of the SC population is indicative that the processes that determine and maintain the cellular organisation of the MTs might extend past embryogenesis in the development of the fly, and that the MTs are not completely developmentally static and might be able to respond to environmental cues to assure tissue homeostasis throughout the life of the animal.

Both SC*^Ssk^*^RNAi^ and PC*^Ssk^*^RNAi^ MTs also had an increased cross-sectional diameter (Fig. 5D), a phenotype iterating pathological changes observed during intestinal barrier dysfunction (Izumi et al., 2019; Rera et al., 2013a; Salazar et al., 2018) and when Mesh or Tsp2A – the obligate partners of Ssk – are impaired in MTs (Beyenbach et al., 2020; Jonusaite et al., 2020). Complementing these observations, although transmission electron microscopy (TEM) scans (Fig. 5G,H) showed that the overall septal junction span was significantly reduced (Fig. 5E), the junctional spans were in fact highly irregular (as evidenced by the frequency distribution in septal junction measurements; Fig. 5F). Importantly, there was a complete absence of septa (Fig. 5G–H′), a consequence of which is loss of paracellular barrier function (Baumgartner et al., 1996; Genova and Fehon, 2003; Lamb et al., 1998). The absence of septa does not itself result in loss of septal-gap structural integrity, as demonstrated by septal mutants such as *sinuous* and *coracle* (Lamb et al., 1998; Wu et al., 2004). Rather, structural integrity is a function of the adherens junctions (AJs) (Tepass and Hartenstein, 1994). Dlg localisation, however, has been shown to be regulated by, and in turn modulate, AJ formation (Bilder et al., 2003, 2000; Bonello, et al., 2019; Harris and Peifer, 2004), so that AJ failure might be an effect of the mislocalisation of Dlg occurring as a consequence of Ssk impairment, which then manifests in the failure of septal-gap structural integrity. It has been shown that AJs are also required for integration, localisation and determination of the polarity of the SC population in the developing tubule (Campbell et al., 2010). Therefore, any compromise of AJs might also contribute to the observed atypical SC distribution, loss of polarity and failure of the junctional complexes.

Loss of septa results in failure of paracellular barriers, which can allow opportunistic toxic and/or pathogenic invasion (Izumi and Furuse, 2014; Salazar et al., 2018). TEM of experimental animals showed evidence of bacterial invasion (Fig. 5H″), which appeared to be localised near the apical (luminal) membrane of affected cells, not observed in controls. This increasing bacterial load could result in chronic activation of inflammatory and/or immune responses (Izumi et al., 2019; Rera et al., 2013a, 2012; Resnik-Docampo et al., 2018; Salazar et al., 2018), again iterating pathological phenotypes that occur during intestinal barrier dysfunction, contributing to reduced organismal viability (Izumi et al., 2019; Rera et al., 2013a; Salazar et al., 2018).

Taken together, these results demonstrate that cell-specific depletion of *Ssk* not only compromises individual cellular junctional complexes, but also affects tubule morphology and organisation as a whole. The finding that impairment of junctional complexes in one restricted sub-population of cells affects overall tissue function supports the observation that non-cell-autonomous communication(s) occur across these ‘tight’ junctions, an observation supported by the findings that mutant gut clonal cells in which *Tsp2A* expression is impaired induced non-cell-autonomous stem cell proliferation (Izumi et al., 2019) and that *mesh* knockdown in the PC population then resulted in dysmorphic SC development (Jonusaite et al., 2020).

### Cell-specific Ssk depletion results in proliferation of tiny cells and hyperplasia of trachea supplying the MTs

We assessed the efficacy of the *Ssk*^RNAi (Furuse)^ transgene via quantitative real-time PCR (qRT-PCR) analysis and immunolocalisation using an antibody specific to Ssk. Despite SCs representing a minority sub-population of cells in the tissue as a whole, both in numbers and in cell size, we were still able to observe a significant tissue-specific knockdown of ∼35% Ssk in 5-day-old adult SC*^Ssk^*^RNAi^ MTs compared with that in controls (Fig. S7A).

**Fig. 6.**
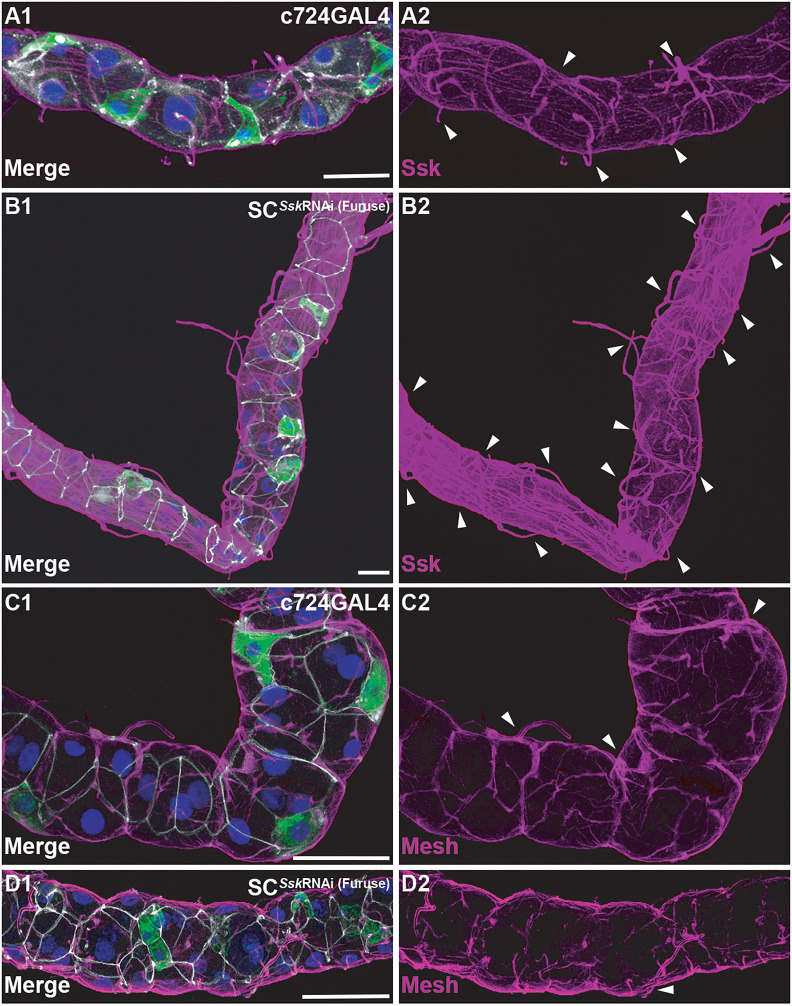
**Cell-specific depletion of *Ssk* results in hyperplasia of trachea supplying adult MTs.** (A1,A2) Representative image of a 5-day-old adult MT with *c724*GAL4 driving mGFP expression, demonstrating control levels of Ssk expression associated with junctions and (at lower levels) trachea. (B1,B2) Overt hyperplasia of trachea, indicated by Ssk expression, associated with the main segment of a SC*^Ssk^*^RNAi (Furuse)^ 5-day-old adult MT. (C1,C2) *c724*GAL4 driving mGFP expression in a 5-day-old adult MT, demonstrating control levels of Mesh expression associated with junctions and (at lower levels) trachea. (D1,D2) A SC*^Ssk^*^RNAi (Furuse)^ 5-day-old adult MT demonstrating a disorganised and reduced pattern of Mesh. All panels are from the main segment of the MT, with overt associated trachea indicated (arrowheads). Note that control and experimental confocal stacks for A,B and B,C were collected using identical settings. mGFP, green; Ssk and Mesh, magenta; Dlg, white; DAPI, blue. Images in A1–D2 are representative of 25 animals. Scale bars: 50 µm.

In SC*^Ssk^*^RNAi^ adults, we observed overt hyperplasia of the trachea supplying the MTs compared to that in controls (Fig. 6A,B; Fig. S7D–J). This hyperplasia of trachea was also apparent in PC*^Ssk^*^RNAi^ MTs, although not to the levels observed with SC*^Ssk^*^RNAi^ MTs (Fig. S7B,C). Immunolocalisation of the anti-Ssk antibody yielded the surprising observation that there appears to be concomitant Ssk localisation to the trachea (Fig. 6B; Fig. S7C). This might be artefactual, resulting from non-specific binding of the antibody to the trachea, or might indicate ectopic expression of Ssk occurring as a result of, or in conjunction with, dysregulation of the associated trachea. It should be noted that application of an antibody specific to Mesh, the obligate partner of Ssk (Izumi et al., 2012), demonstrated that distribution of Mesh at the junctional complexes appeared reduced and disorganised in SC*^Ssk^*^RNAi^ MTs, but did not manifest the same increased expression in the associated trachea (Fig. 6C,D).

In both SC*^Ssk^*^RNAi^ and PC*^Ssk^*^RNAi^ MTs, we also observed an increased number of nuclei associated with tiny cells in the proximal area of the tubule and ureter (Fig. S8). These tiny cells, described as renal nephritic stem cells (RNSCs) in healthy young animals, are quiescent and confined to the lower tubule or ureter region (Cohen et al., 2020; Martinez-Corrales et al., 2019; Singh and Hou, 2009, 2008; Singh et al., 2011; Wang and Spradling, 2020), but have been observed to increase in number in response to stress or as MTs degenerate over time. It is therefore of interest that in SC*^Ssk^*^RNAi^ tubules, this cell population was significantly increased along the proximal segment, with 117.5±21.78 (mean±s.d.) cells in the first 300 µm of the tubule beyond the ureter compared to 53.7±11.26 cells in controls, whereas there appeared to be no significant increase in cell numbers in the ureter itself in experimental animals compared with those in controls (Fig. S7). In keeping with the description that these cells are RNSCs, some cells were observed to co-express the proliferative cell markers Delta (Biteau et al., 2008) and Hindsight (also known as Pebbled) (Bohère et al., 2018) (Fig. S7P–S). However, we cannot rule out the possibility that this might be anomalous expression of proliferative markers as a consequence of epithelial dysfunction, as has been observed to occur during gut barrier dysfunction when some intestinal enterocytes express intestinal stem cell (ISC)-specific proliferative markers (Resnik-Docampo et al., 2017).

### Ssk impairment accelerates age-related changes in tubule SJ integrity and secretory capacity

**Fig. 7.**
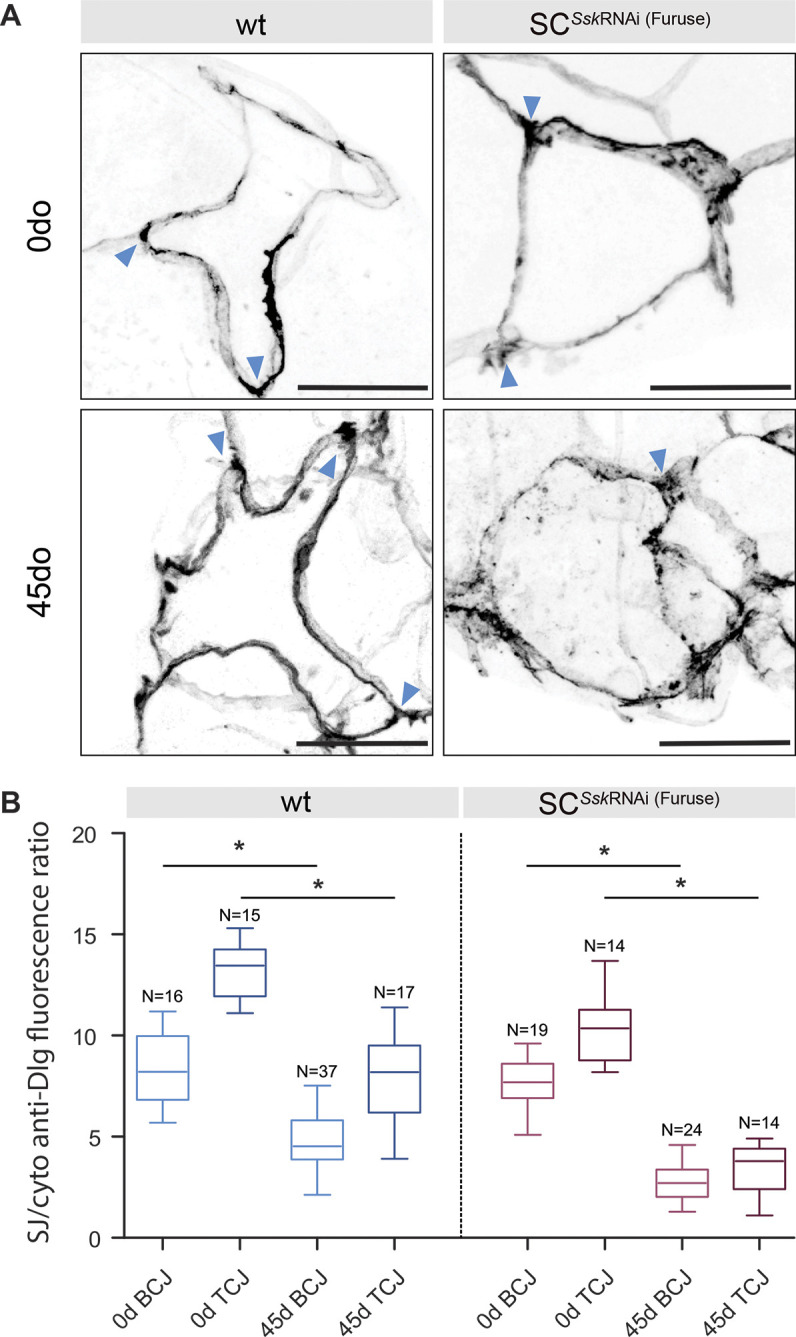
**SC-specific depletion of *Ssk* impairment advances age-related changes in junctional protein localisation.** (A) Distribution of Dlg in adult wild-type (wt) and SC*^Ssk^*^RNAi^ SCs at 0 and 45 days post eclosion. Tricellular junctions are indicated by arrowheads. Scale bars: 20 μm. (B) Graphical representation of the ratio of Dlg fluorescence signal at bicellular (BCJ) and tricellular (TCJ) junctions compared with cytoplasmic signals in adult wt and SC*^Ssk^*^RNAi^ SCs at 0 and 45 days post eclosion. Boxes show the 25–75th percentiles, whiskers show the minimum and maximum values, and the median is marked with a line. *P<0.0001, two-tailed unpaired Student's *t*-test.

**Fig. 8.**
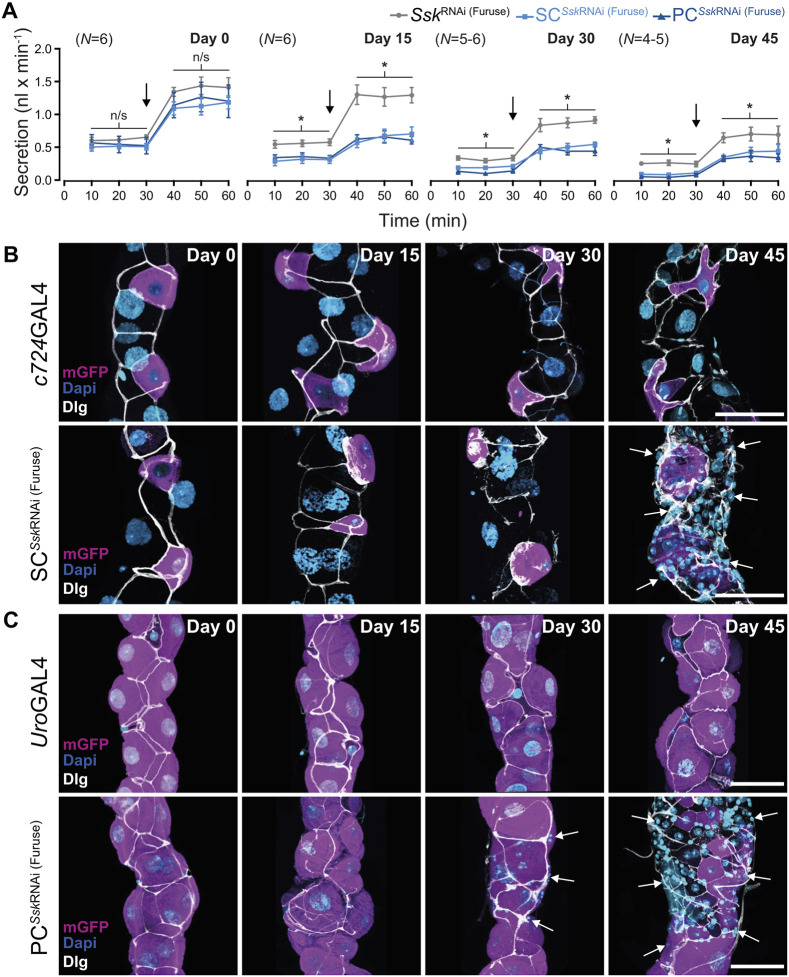
**Ssk impairment accelerates age-related changes in SJ integrity and secretory capacity in adult MTs.** (A) Modified Ramsey assay measuring fluid secretion rates in control and experimental adult MTs over time as flies aged. The secretion rate capacity for all tubules (both control and experimental) decreased progressively over time. Secretion rates post day 15 for SC*^Ssk^*^RNAi (Furuse)^ and PC*^Ssk^*^RNAi (Furuse)^ MTs were significantly reduced compared to those of controls, even after stimulation with DromeKinin (black arrows). Data shows the mean±s.e.m. *P<0.05, two-tailed paired-samples *t*-test. Representative images of the accelerated degeneration of tubule morphology and mislocalisation of Dlg in SC*^Ssk^*^RNAi (Furuse)^ (B) and PC*^Ssk^*^RNAi (Furuse)^ (C) MTs compared with those in controls. Post-day 30 proliferation of tiny cells in SC*^Ssk^*^RNAi (Furuse)^ and PC*^Ssk^*^RNAi (Furuse)^ MTs overt (arrows) is indicated. mGFP, magenta; Dlg, white; DAPI, blue. Images in B,C are representative of 25 animals. Scale bars: 50 µm.

To assess potential age-related changes in tubule physiology, we examined junctional integrity by analysing Dlg localisation as well as renal secretory capacity, at progressive timepoints during ageing. We observed a significant decrease in Dlg intensity associated with cell–cell junctions in older animals, compared with that in younger animals (Fig. 7), as well as a progressive decline in secretory activity, as evidenced by significant reductions in both basal and stimulated rates of tubule secretion (Fig. 8A; Fig. S8). These data suggest that junctional stability declines with age and that there is a measurable ‘natural’ deterioration in the transport capacity of the tubule that correlates with physiological ageing (Fig. 8A; Fig. S8A).

We then investigated whether compromising junctional integrity through cell-specific depletion of *Ssk* could phenocopy these age-related changes in tubule function. Knocking down Ssk expression in either SC*^Ssk^*^RNAi^ or PC*^Ssk^*^RNAi^ MTs resulted in an accelerated and progressive decline in tubule secretory capacity relative to that in age-matched controls (Fig. 8; Fig. S8). This decline was evident as early as 15 days post eclosion, with this decrement in function mirrored by an associated degeneration in tissue morphology, with overt signs of apoptosis, mislocalised cytoplasmic Dlg accretions and a dramatic increase in tiny cells (Fig. 8B,C; Fig. S7). Compellingly, when comparing secretory rates between 45-day-old control and 15-day-old experimental animals, although the basal secretory rate of the experimental animals was marginally better, there was no significant difference in stimulated rates of tubule secretion between control and experimental animals (Fig. S8B). For example, 15-day-old experimentally aged tubules recapitulated the reduced secretory capacity of aged 45-day-old controls. Finally, the fact that compromised junctional integrity resulted in an accelerated loss in tissue homeostasis is supported by the observed significant increase in Dlg mislocalisation in SC*^Ssk^*^RNAi^ MT cellular junctions compared with that in controls of the same age (Fig. 7A,B). Taken together, these data demonstrate that the tubule undergoes an age-dependent decline in tissue homeostasis and transport capacity, suggesting that compromised barrier function is an import factor underpinning ageing.

## DISCUSSION

Transporting epithelia must be able to proliferate appropriately in response to growth directives (organogenesis, wound response, etc.), while maintaining cellular compartmentalisation to ensure that key systems are protected from physiological and xenobiotic stresses. Concomitantly, they need to provide a physical barrier against invasive pathogens, while allowing appropriate transport of ions, solutes and water. Pivotal to these diverse functional demands is the formation and maintenance of tight cell–cell junctional complexes. The profound consequences associated with a failure in these processes have been demonstrated in intestinal epithelia dysfunction, resulting in physiological decline contributing to onset of age-related disease and, ultimately, death (Clark and Walker, 2018; Clark et al., 2015; Davies et al., 2012; Hu and Jasper, 2017; McGee et al., 2011; Regan et al., 2016; Rera et al., 2013a, 2012; Resnik-Docampo et al., 2018, 2017; Salazar et al., 2018).

### Ssk depletion phenocopies the natural age-related changes in tubule junctional integrity that affect organismal viability

Our investigations demonstrate that, as flies age, there occurs a measurable natural decline in tubule transport capacity. This occurs in conjunction with, or as a result of, mislocalisation of cellular junctional components, indicative of a failure in cell–cell junctional integrity, all of which manifest in a progressive decline in organismal homeostatic capabilities and viability. This is supported by Salazar et al. (2018), who demonstrated that inflammatory response genes are naturally upregulated as flies age and that intestinal barrier function was equally compromised when comparing controls at day 45 with experimental flies in which intestinal barrier dysfunction had been induced.

We demonstrated that restrictively impairing the essential junctional protein Ssk (Yanagihashi et al., 2012) in either SCs or PCs both phenocopies and advances normal degenerative changes, affecting both individual cell populations and overall tissue morphology. These effects resulted in a measurable accelerated decline in the physiological capacities of the tubules, culminating in significantly reduced organismal viability.

### Ssk depletion promotes failure of the junctional complex, resulting in loss of cellular cytoarchitecture and apicobasal polarity

The occluding junction proteins Ssk and Tsp2a and the cell adhesion protein Mesh are mutually dependent for their localisation to sSJs and critical for proper formation of the sSJs (Beyenbach et al., 2020; Jonusaite et al., 2020; Izumi et al., 2012, 2016; Salazar et al., 2018; Xu et al., 2019; Yanagihashi et al., 2012). This Mesh–Ssk–Tsp2a complex creates a platform for appropriate assembly and localisation of other key junctional proteins (Beyenbach et al., 2020; Jonusaite et al., 2020; Izumi et al., 2012, 2016; Salazar et al., 2018; Xu et al., 2019). Although in ectodermally derived epithelial pSJs, Dlg is not a core junctional component, its misregulation still dramatically affects junctional complexes (Oshima and Fehon, 2011; Izumi et al., 2012). In keeping with this, although Mesh, Ssk and Tsp2a are able to localise to sSJs independently of Dlg (Yanagihashi et al., 2012; Izumi et al., 2012, 2016), the individual junctional components are essential for appropriate localisation of Lgl (Jonusaite et al., 2020; Izumi et al., 2012, 2016; Yanagihashi et al., 2012). As Lgl and Dlg have a mutually dependent functional relationship (Bilder et al., 2000, 2003; Izumi et al., 2012; Su et al., 2012), if, in the absence of Ssk, Lgl is mislocalised at the apical membrane of the MTs, this would result in the observed mislocalisation of Dlg, as well as affecting other components of the sSJs (Beyenbach et al., 2020; Jonusaite et al., 2020; Izumi et al., 2019). This misregulation of Dlg, evidenced by progressive formation of cytoplasmic accretions, also results in disruption of microfilament networks responsible for the internal cellular cytoarchitecture (Bilder and Perrimon, 2000; Bilder et al., 2003; Tanentzapf and Tepass, 2003; Woods et al., 1996; Wu and Beitel, 2004; Yu and Fernandez-Gonzalez, 2016), most evident in the dysmorphic SCs in which F-actin appears entirely absent.

Dlg, Lgl and Scribble constitute the Scribble polarity module, a regulator of cell polarity and proliferation (Bonello et al., 2019; Elsum et al., 2012; Humbert et al., 2008, 2003; Khoury and Bilder, 2020), with mislocalisation of Dlg resulting in loss of apicobasal polarity (Bilder and Perrimon, 2000; Bilder et al., 2003; Bonello et al., 2019; Tanentzapf and Tepass, 2003; Woods et al., 1996; Wu and Beitel, 2004). In SC*^Ssk^*^RNAi^ animals, this is evidenced by mislocalisation of the SC-specific basolateral Prip and apical Drip aquaporin channels, and the absence of the basolateral chloride (ClC-a) channel (Cabrero et al., 2014). Loss of the ClC-a channel, coupled with mislocalisation of the aquaporin channels, means that the Cl^−^ shunt, necessary to create the osmotic gradient required for water flux (Cabrero et al., 2020, 2014; Denholm et al., 2013), is compromised, again contributing to the decrement in osmoregulatory capacity.

### Compromised osmoregulatory and barrier integrity in the tubule epithelium is a consequence of failure of septa

Loss of septa leads to a failure in paracellular barrier integrity, which would contribute in large part to the compromised fluid transport capabilities of MTs, with the compromised osmoregulatory and barrier integrity in tubule epithelium again phenocopying effects observed with age-related loss of intestinal integrity (Rera et al., 2012; Resnik-Docampo et al., 2017, 2018; Salazar et al., 2018). This loss of barrier function could allow opportunistic invasion of pathogens, such as the bacterium observed to be associated with the apical membrane in Ssk^RNAi^ tubules, leading to dysbiosis and activation of immune and/or inflammatory responses (Clark and Walker, 2018; Clark et al., 2015; Izumi and Furuse, 2014; Rera et al., 2013b; Resnik-Docampo et al., 2018; Salazar et al., 2018; Xu et al., 2019).

Concomitant with loss of septa was an observed irregularity of junctional spans, indicating a loss in junctional structural integrity derived from the AJs (Tepass and Hartenstein, 1994). AJs provide cues for appropriate localisation of, and might then be modulated by, Dlg in junctional complexes (Bilder et al., 2000, 2003; Bonello et al., 2019; Harris and Peifer, 2004) and are also required during the integration of the SCs in the developing tubule for proper localisation and polarity of the SC population during formation of junctional complexes (Campbell et al., 2010). Any compromise of these structures might explain the observed atypical distribution, loss of polarity and failure of the junctional complexes in the SC population. Furthermore, the fact that increased clustering of SCs occurs when *Ssk* expression is knocked down in pupal and adult stages indicates that the MTs are not developmentally static and are able to respond to environmental cues to ensure tissue homeostasis in the adult.

### Cell-specific depletion of Ssk in the tubule epithelium has global consequences on tissue function and organismal viability

The notion that impairment of junctional complexes in a specific sub-population of cells affects overall tissue function and morphology implies that non-cell-autonomous communication(s) are required across these tight junctions. This is supported by the observations that mutant clonal cells in the gut in which *Tsp2A* expression is impaired induced non-cell-autonomous stem cell proliferation (Izumi et al., 2019) and that *mesh* knockdown in PCs also resulted in dysmorphic SC development (Jonusaite et al., 2020).

The apparent dysregulation of Dlg, a known tumour suppressor gene (Elsum et al., 2012; Humbert et al., 2008, 2003), in individual junctional complexes might also speak to the effects that occur more universally throughout the mutant MTs. Polarity proteins have been shown to directly modulate signalling pathways controlling tissue growth (Royer and Lu, 2011; Tepass et al., 2001) and certainly some of the most striking global effects observed in both SC*^Ssk^*^RNAi^ and PC*^Ssk^*^RNAi^ MTs are the proliferation of the RNSCs and the hyperplasia that occurs in the associated trachea supplying the MTs. Whether the tracheal hyperplasia is an indirect effect of local tissue hypoxia due to a compensatory upregulation of metabolic function, as seen during fly development (Texada et al., 2019), or a direct consequence of impairment of appropriate tissue growth regulation due to dysregulation of the junctional complexes warrants further investigation. However, the proliferation of the RNSCs (Martínez-Corrales et al., 2019; Singh and Hou, 2008, 2009; Singh et al., 2011; Wang and Spradling, 2020), extending beyond their normal distribution pattern from the ureter into the proximal and main segments, appears to occur in direct response to the progressive failing of the physiological capacity of the tubules. Although these RNSCs appear proliferative, there is the possibility that they are anomalously expressing proliferative markers, as was observed with enterocyte-like cells expressing ISC-specific proliferative markers during intestinal barrier dysfunction (Resnik-Docampo et al., 2017). However, it is not unreasonable to relate these effects, occurring in conjunction with or as a result of loss of barrier function allowing opportunistic pathogenic challenge and consequent dysbiosis, to the inflammatory and ageing responses involving the JNK and Jak/Stat/cytokine signalling pathways that direct increased proliferation of stem cells and other regenerative homeostatic processes in the gut (Biteau et al., 2008; Izumi et al., 2019; Jiang et al., 2009; Rera et al., 2012; Resnik-Docampo et al., 2017, 2018).

In this study, we demonstrate a measurable age-dependent decline in secretory transport capacity in insect renal tubules, associated with mislocalisation of smooth SJ proteins and coincident progressive degeneration in associated cellular and tissue morphology. Critically, by cell-specific depletion of *Snakeskin*, we were able to phenocopy the failure of junctional complexes, leading to an accelerated reduction in secretory capacity. Our work thus introduces the *Drosophila* tubule as a powerful model for studies of age-related loss of homeostasis in a classically non-proliferative tissue; a model that allows insight into the mechanisms by which failure in epithelial barrier integrity can advance loss of systemic fluid homeostasis, resulting in accelerated morbidity and, ultimately, death.

## MATERIALS AND METHODS

### *Drosophila* stocks

*Drosophila* lines were reared on standard Glasgow *Drosophila* diet at 45–55% relative humidity with a 12 h:12 h light:dark photoperiod at a temperature of 22°C, unless otherwise stated. Parental (control) and F1 progeny (experimental) strains expressing the *tubPGAL80^ts^* transgene were raised at the permissive temperature (18°C), before the F1 (experimental) progeny were transferred at late L3 or white pre-pupal stage to the restrictive temperature (29°C), unless otherwise stated*.* Where possible, control animals were also moved to the restrictive temperature (29°C) to ensure matching the developmental age of the experimental flies.

The w*; *Sco/CyO*; *tubP GAL80^ts^* (7018), w*; tubP GAL80^ts^/*TM2* (7017), w*; tubP GAL80^ts^; *TM2/TM6b Tb^1^* (7108), y^1^ w*; Pin^Yt^/*Cyo*; UAS-*mCD8::GFP* (5130) and y^1^ w*; UAS-*mCD8::GFP* (5137) lines were acquired from the Bloomington *Drosophila* Stock Center (Bloomington, IN, USA). The *w^1118^; ClC-a*GAL4; (202625), w*; UAS-*Ssk*^RNAi (GD)^; (11906) and w*;; UAS-*Ssk*^RNAi (KK)^ (105193) lines were acquired from the Vienna *Drosophila* Resource Center (Vienna, Austria). The w*;; UAS-*Ssk*^RNAi (Furuse)^ line (Yanagihashi et al., 2012) was a kind gift from the Furuse laboratory (National Institute for Physiological Sciences, Okazaki, Japan). The *c724*GAL4 (Sözen et al., 1997) and *Uro*GAL4 (Terhzaz et al., 2010) lines were previously generated in-house.

### Immunocytochemistry

Unless otherwise stated, adult flies were reared at 29°C and aged for 5 days prior to dissection in Schneider's *Drosophila* medium (Gibco, Thermo Fisher Scientific). Immunocytochemistry staining was performed as previously described (Halberg et al., 2015). The following primary antibodies were used: mouse monoclonal anti-Na^+^/K^+^ vATPase α-subunit (1:50, a5), rabbit polyclonal anti-Dcp-1 (1:100, cat. no. 9578) from Cell Signaling Technology Europe (Leiden, The Netherlands); anti-Dlg1 (1:500, 4F3), anti-Delta (1:200, C594.9b) and anti-Hnt (1:30, 1G9), all from Developmental Studies Hybridoma Bank (University of Iowa, IA, USA); rabbit polyclonal anti-Snakeskin (1:1000) and anti-Mesh (1:1000) from the Furuse laboratory (Izumi et al., 2012; Yanagihashi et al., 2012); rabbit polyclonal anti-ClC-a (1:50) (Cabrero et al., 2014), anti-Drip (1:1000) and anti-Prip (1:1000) (Cabrero et al., 2020; from our laboratory). The following secondary antibodies were used: Alexa Fluor 488- or 546-conjugated anti-rabbit IgGs (cat. no. A-11034 and A-11035, respectively) and Alexa Fluor 633-conjugated anti-mouse IgG (cat. no. A-21052) (all at 1:600; Thermo Fisher Scientific). Tubules were also incubated in 500 ng/ml DAPI and Phalloidin::TRITC (Sigma-Aldrich). Tissues were mounted on either Polysine slides (VWR International, Leuven, Belgium) or glass-bottomed dishes (MatTek Corporation, MA, USA) and analysed using a Zeiss LSM 880 confocal micro-system (Carl Zeiss, Cambridge UK). Confocal *z*-projection stacks used for cell counts, analyses and presentation were opened in ImageJ (National Institutes of Health, MA, USA) prior to transfer to Adobe Photoshop and Illustrator (CS6; CA, USA) for final presentation.

### TEM

Tubules were dissected from control and experimental adult flies and fixed in trialdehyde consisting of 2.5% glutaraldehyde, 2% paraformaldehyde, 1.25% acrolein and 2.6% DMSO in 0.1 M sodium cacodylate buffer (pH 7.4) overnight at 4°C. The tissues were post fixed in 1% OsO_4_ buffered with 0.1 M sodium cacodylate adjusted to pH 7.4 for 1 h at room temperature. Next, the tubules were dehydrated through a graded series of ethanol and propylene oxide, before being embedded in epoxy resin EPON 812 (TAAB, Berkshire, UK). Ultrathin sections were cut with a diamond knife on a Leica UTC ultramicrotome and stained with half-saturated (2%) uranyl acetate, followed by Reynolds' lead citrate. The ultrathin sections were examined in a FEI Tecnai T20 electron microscope.

### RNA isolation, cDNA synthesis and qRT-PCR

RNA was isolated from 30 pairs of dissected adult tubules using TRIzol Reagent (Thermo Fisher Scientific) resuspended in nuclease-free dH_2_O. cDNA was synthesised from 500 ng RNA using SuperScript II RT (Thermo Fisher Scientific), following the manufacturer's instructions. qRT-PCR was performed using Brilliant III SYBR Green QPCR Master Mix (Agilent) on the StepOne+ Real-Time PCR system (Thermo Fisher Scientific) using primers specific to *snakeskin* (SskF1: 5′-TTACACTGGATGCCACACCATTGC-3′; SskR1: 5′-TGACGCTCCGAGTTCACATACAGG-3′) and *α-tubulin 84b* (TubF1: 5′-CCTCGAAATCGTAGCTCTACAC-3′; TubR1: 5′-ACCAGCCTGACCAACATG-3′) (Integrated DNA Technologies). Following amplification, the StepOne software was used to generate a standard curve. The relative concentration was determined by placing the cycle threshold (Ct) value and the values from the gene standard onto the standard curve. Each sample was then normalised against α-tubulin, resulting in a ratio of gene/α-tubulin expression. Results were then plotted as mean mRNA amounts (±s.e.m.) using Prism 6.0 (GraphPad, CA, USA).

### Fly weight measurements

To measure wet-body weight, 20 female flies (*n*=8–19 groups) were anesthetised, transferred to pre-weighed Eppendorf tubes and weighed. For dry-body weight, flies were killed by freezing for 20 min and then dried at 60°C for 48 h. Dry flies were weighed after reaching room temperature. All weights were measured using a GR-202 analytical balance (A&D Instruments, Abingdon, UK).

### Survival assays

All survival assays comprised a minimum of 180 flies (*n*=30 flies per vial, three vials per sex) and were performed in 12 h:12 h light:dark ratio at the restrictive temperature (29°C). Starvation assay vials contained 7 ml of 1% low melting point (LMP)-agarose in water. Osmotic stress assay vials contained 7 ml of standard fly medium with the addition of 3% NaCl. The age of the flies ranged between 3 and 5 days. Data for all assays were plotted as Kaplan–Meier curves and analysed using the Mantel–Cox (log-rank) test using Prism v6.0 (GraphPad, CA, USA).

### Ramsay fluid secretion assay

Fluid secretion assays using *Drosophila* MTs were performed as described previously (Halberg et al., 2015; Dow et al., 1994). MTs were dissected in ice-cold Schneider's medium and transferred to a 9 µl drop of 1:1 of Schneider's medium and *Drosophila* saline containing trace amounts of amaranth (cat no. A1016; Sigma-Aldrich). After an acclimatisation period, baseline secretion rates were measured every 10 min for 30 min, after which 1 µl of DromeKinin peptide (10^−6^ M; Cambridge Peptides, UK) (Terhzaz et al., 1999) was added to the drop and secretion was measured at 10 min intervals for a further 30 min. An increase in fluid secretion rate following DromeKinin application compared to unstimulated basal conditions was taken as an indication of a diuretic effect.

## Supplementary Material

10.1242/joces.261118_sup1 Supplementary information
