# Supplementary material for: Compromised junctional integrity phenocopies age-dependent renal dysfunction in Drosophila Snakeskin mutants
Source: J Cell Sci. 2023 Oct 5;136(19):jcs261118. doi: 10.1242/jcs.261118 (PMC10565245; doi:10.1242/jcs.261118)
Supplement: Supplementary information [file joces-136-261118-s1.pdf]

Figure S1.

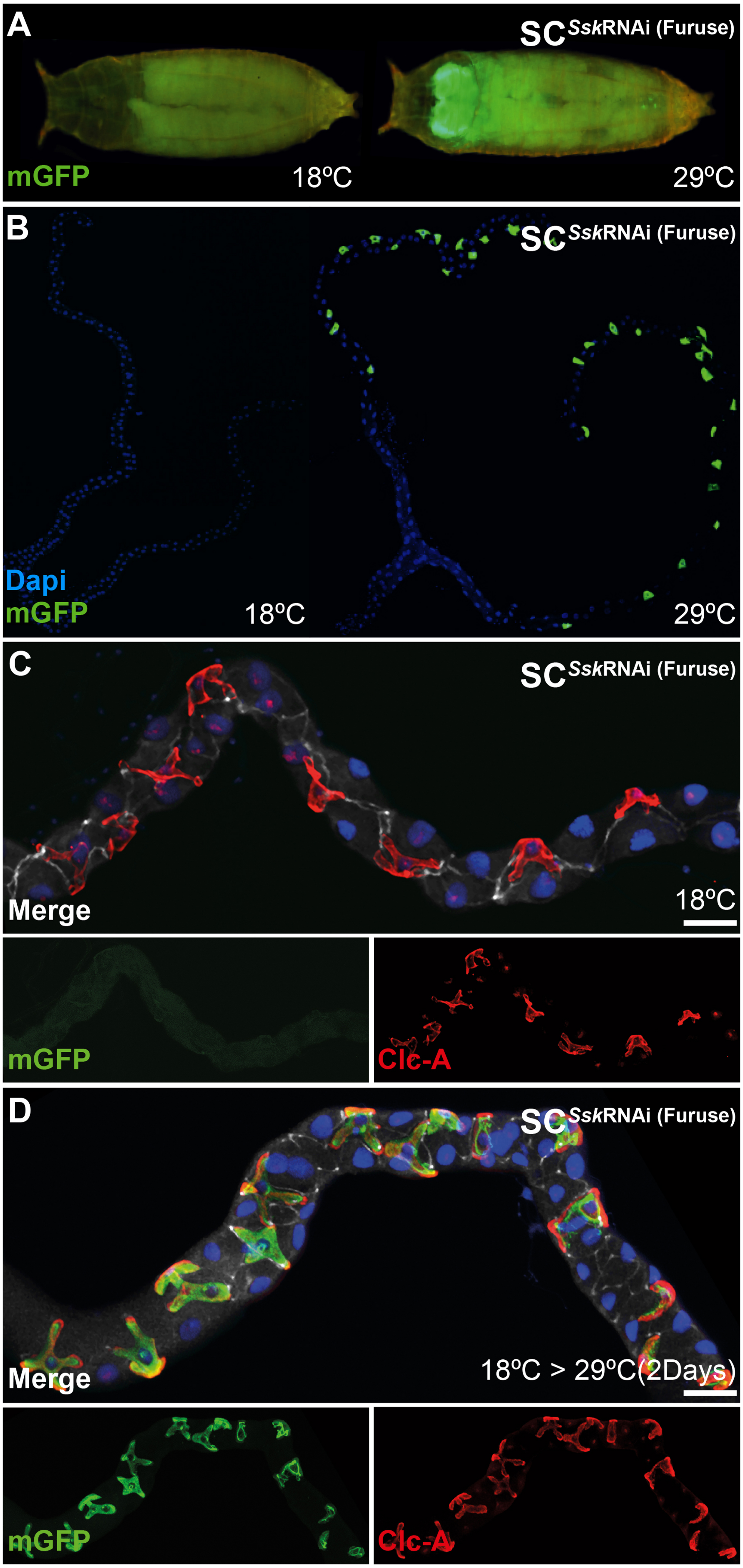
A
SC SskRNAi (Furuse)
mGFP
18ºC
29ºC
B
SC SskRNAi (Furuse)
Dapi
mGFP
18ºC
29ºC
C
SC SskRNAi (Furuse)
Merge
18ºC
mGFP
Clc-A
D
SC SskRNAi (Furuse)
Merge
18ºC > 29ºC(2Days)
mGFP
Clc-A

Fig. S1. Validation of either repression or expression of transgene reporters at the permissive (18ºC) and restrictive (29ºC) temperatures. (A) Membrane-bound green fluorescent protein (mGFP) expression in 24 hr pupae; absent at 18ºC (permissive temperature) but evident at 29ºC (restrictive temperature). (B) mGFP expression in 5 d adult tubules; absent at 18ºC (permissive temperature) but present in tubules of adults raised at 18ºC and then transferred to 29ºC (restrictive temperature). (C) $SC^{\mathit{Ssk}\mathrm{RNAi}}$ 5 day adult tubule raised at 18ºC (permissive temperature). mGFP expression is completely absent, however staining with SC-specific Clc-A antibody reveals SC population normal both for morphology (mature stereotypical stellar appearance) and distribution.

Figure S2.

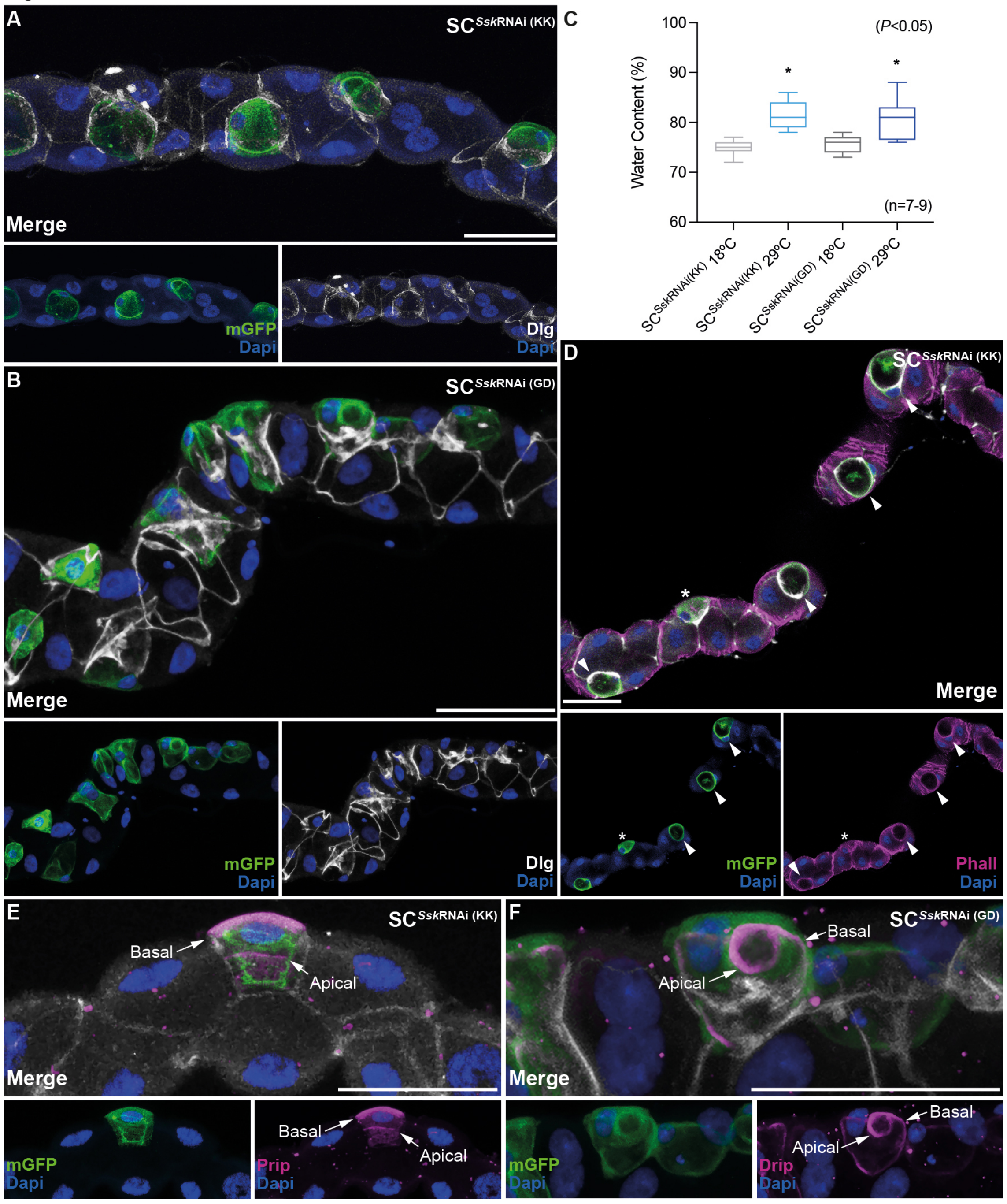

Fig. S2. SC-specific depletion of Ssk using alternative independent RNAi lines in adult Malpighian tubules iterating observed compromised cellular and junctional morphology.

(A) 5 day adult SC$^{Ssk\text{RNAi (KK)}}$ MT. SCs exhibiting absence of mature stellar morphology, appearing vacuolar and extruded from the plane of the MT. Junctional complexes, as realised by anti-discs large (white), appear disorganised or missing with accretions associated with SCs. (B) 5 day adult SC$^{Ssk\text{RNAi (GD)}}$ MT. SCs, clustered within the MT, range from those displaying some stellar-like morphology to those exhibiting complete absence of mature stellar morphology. The more compromised SCs again appear vacuolar and extruded from the plane of the MT. Junctional complexes, as realised by anti- discs large (white) appear disorganised or missing with accretions associated with the SCs. (C) Demonstration of significant difference for water content between experimental (29ºC) SC$^{Ssk\text{RNAi (KK)}}$ and SC$^{Ssk\text{RNAi (GD)}}$ females versus (18ºC) controls. Box-plot whiskers, min to max. *P<0.05, Student's T-Test, N's in parentheses. (D) z-stack subset of a 5 day adult SC$^{Ssk\text{RNAi (KK)}}$ MT detailing internal cytoarchitecture of compromised SCs. Cuboidal SCs (arrowheads) exhibit absence of internal cytoarchitecture as realised by Phalloidin-staining of F-actin (Phall). Single MT exhibiting stellar-like morphology (asterisk) appears to demonstrate the presence of some internal F-actin staining. (E) z-stack subset of a 5 day SC$^{Ssk\text{RNAI}}$ (KK) adult MT, demonstrating Prip expression to the basolateral membrane (Basal, arrow) but also mislocalised expression to the apical membrane (Apical, arrow). (F) z-stack subset of a 5 day SC$^{Ssk\text{RNAI (GD)}}$ adult MT, demonstrating Drip expression to the apical membrane (Apical, arrow) but also mislocalised expression to the basolateral membrane (Basal, arrow). mGFP, green; Dlg, white; Phalloidin (C), Prip (E) or Drip (F), magenta; Dapi, blue. Scale bars = 50 μm.

Figure S3.

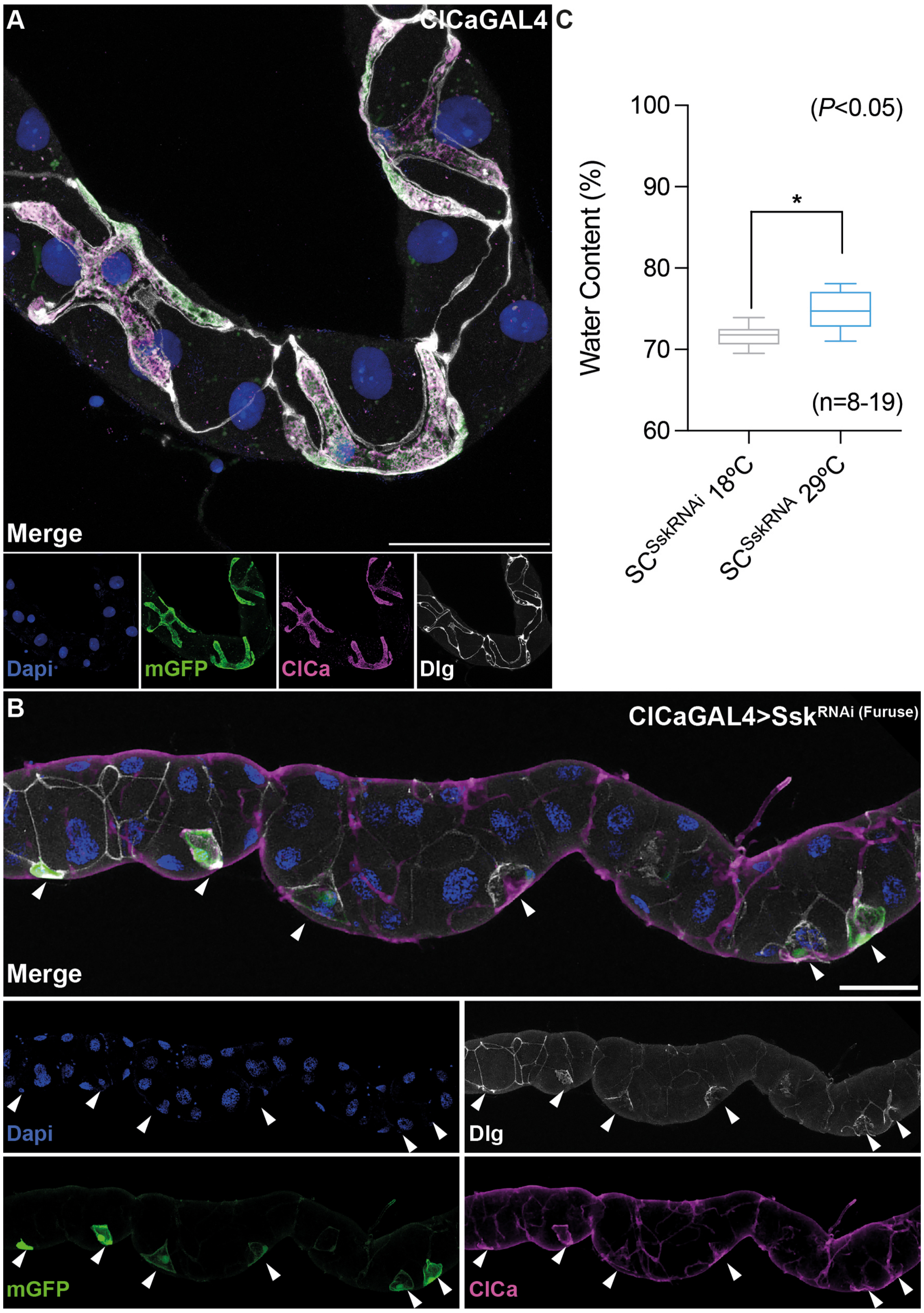

Fig. S3. ClC-aGAL4 knockdown of Ssk in adult Malpighian tubules. (A) ClC-aGAL4 driving membrane-bound (mGFP) in 5 day adult MT, with expression in evenly spaced SCs exhibiting stereotypical stellar morphology, and smoothly organised junctions throughout realized by anti-discs large (Dlg). SCs are stained with anti-ClC-a demonstrating specific localisation to the SC basolateral membrane. (B) ClC-aGAL4 driving $Ssk^{RNAi\ (Furuse)}$ and mGFP in 5 day adult MT. SCs exhibiting absence of mature stellar morphology (arrowheads). Junctional complexes, as realised by Dlg staining, appear disorganised or missing and the tubule itself is distended. ClC-a staining is largely absent, what staining remains is disorganised with absence of localisation to the basolateral membrane indicative of loss of apicobasal polarity in the mutants cells. (C) Demonstration of significant difference between ClC- aGAL4>$Ssk^{RNAi\ (Furuse)}$ experimental (29ºC) flies versus (18ºC) controls for water content, indicative of accumulation of haemolymph. Box-plot whiskers, min to max. *P<0.05, Student's T-Test, N's in parentheses. Dapi, blue; mGFP, green; ClC-a, magenta; Dlg, white. Scale bars = 50 μm.

Journal of Cell Science • Supplementary information

Figure S4.

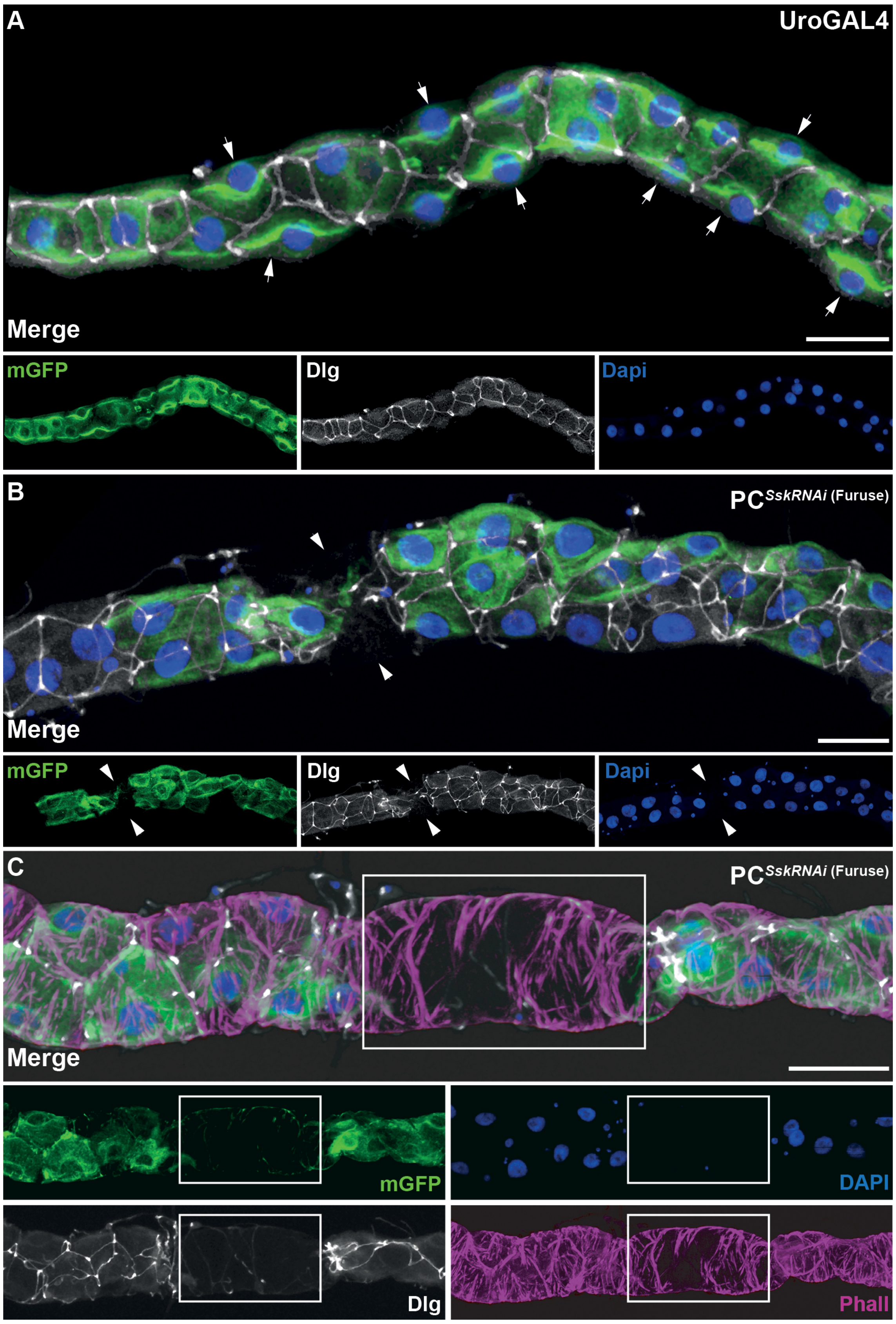

Fig. S4. Cell-specific depletion of *Ssk* in adult Malpighian tubules results in loss of cellular, junctional and cytoarchitectural organisation. (A) *Uro*GAL4 driving membrane-bound green fluorescent protein (mGFP) in 5 day adult MT. mGFP in PCs exhibiting apical bias of expression (arrows), with smoothly organised junctions throughout realized by anti-discs large (Dlg). (B) 5 day experimental adult PC$^{Ssk\text{RNAi}}$ MT. PCs exhibit disorganised, or missing, junctional complexes and apparent loss of apical bias in mGFP expression. Complete absence of staining indicating apoptotic cells indicated (arrowheads). (C) 5 day experimental adult PC$^{Ssk\text{RNAi}}$ MT exhibiting absence of cellular architecture, realised by Phalloidin (Phall) staining as well as junctional expression, mGFP and Dapi staining (white box). mGFP, green; Phall, magenta; Dlg, white; Dapi, blue. Scale bars = 50 µm.

Journal of Cell Science • Supplementary information

Figure S5.

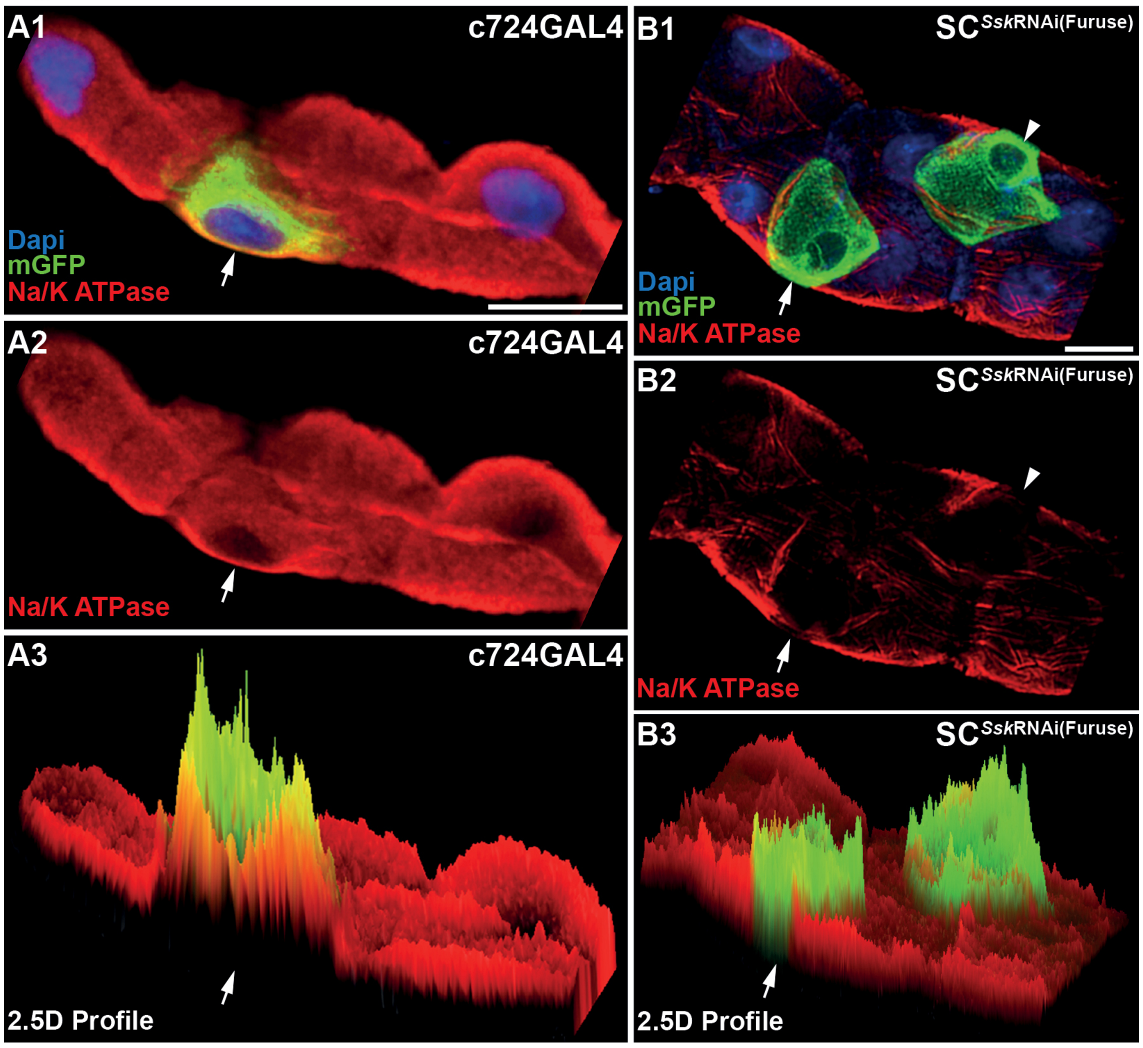

Fig. S5. cell-specific depletion of *Ssk* in adult Malpighian tubules results in loss of apicobasal polarity marker.

(A) Subset of z-stack of *c724*GAL4 driving membrane-bound green fluorescent protein (mGFP) in 5 day adult MT. A1, merged image of confocal stack subset, exhibiting the basal staining for Na+/K+ alpha subunit (Na/K ATPase; arrow). A2, Na/K ATPase alone, highlighting expression at basal cell boundaries. A3, 2.5D rendering of A1 to illustrate Na/K ATPase expression biased to the basal cell boundary of SC (arrow). (B) Subset of z-stack of 5 day adult $SC^{\mathit{Ssk}\mathrm{RNAi}}$ MT. B1, merged image of confocal stack subset, detailing basal edge of SC exhibiting absence of staining for Na/K ATPase at the basal cell boundary (arrow). Second SC with absent Na/K ATPase at the basal cell boundary (arrowhead). B2, Na/K ATPase alone highlighting presence or absence of expression at basal cell boundaries (arrow and arrowhead). B3, 2.5D rendering B1 to illustrate absence of Na/K ATPase expression specific to mutant SC as compared with surrounding principal cells (arrow). 2.5D rendering - intensity values in a 2D image are converted into a height map, with highest intensity values represented by the greatest extension in the Z direction. Note- both confocal stacks were collected using identical settings. mGFP, green; Na/K ATPase, red; Dapi, blue. Scale bars = 20 μm.

Journal of Cell Science • Supplementary information

Figure S6.

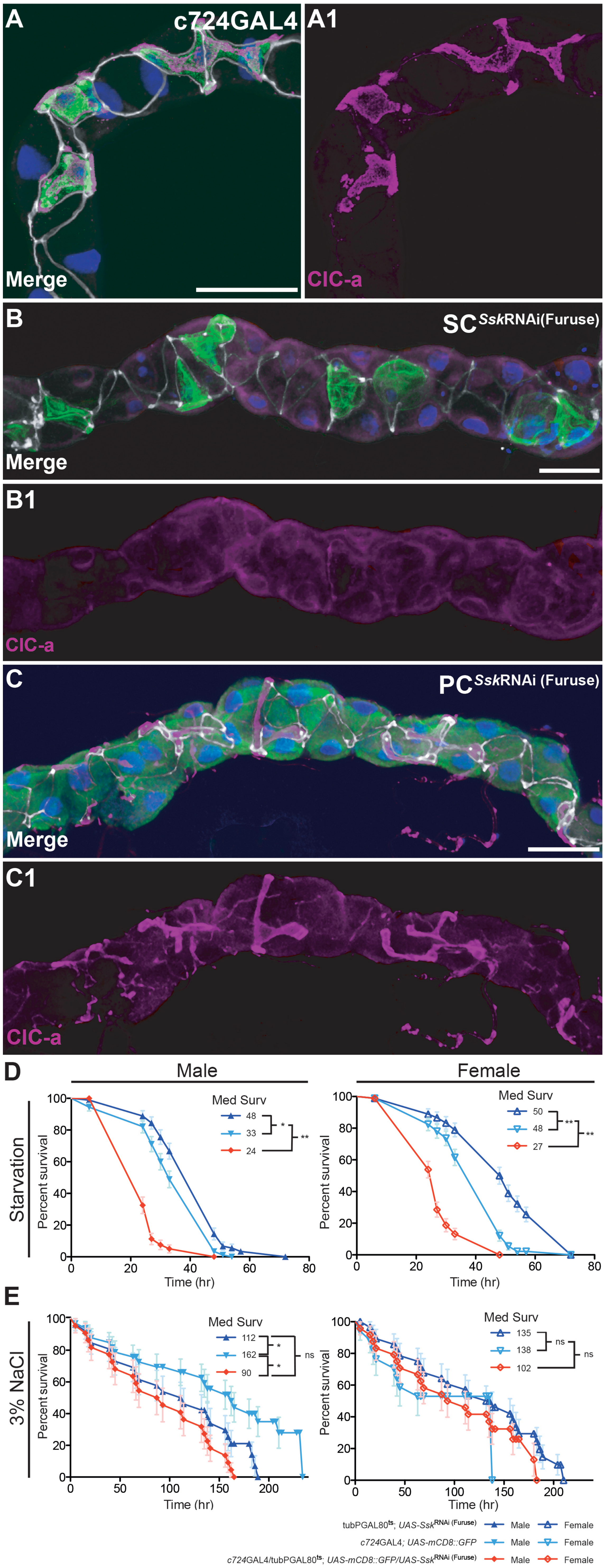

Journal of Cell Science • Supplementary information

Fig. S6. SC-specific depletion of *Ssk* in adult Malpighian tubules results in loss of the SC-specific chloride channel-a with increased sensitivity to starvation but not salt stress conditions.

(A) *c724*GAL4 driving membrane-bound green fluorescent protein (mGFP) in 5 day adult MT, demonstrating co-expression with SC-specific anti-Chloride Channel-a (ClC-a). (B) 5 day adult $SC^{\textit{Ssk}\text{RNAi}}$ MT. SCs exhibit absence of SC- specific ClC-a expression. (C) 5 day adult $PC^{\textit{Ssk}\text{RNAi}}$ MT with with unaffected expression of ClC-a in SCs. Note SC stellar morphology is also unaffected. mGFP, green; ClC-a, magenta; Dlg, white; Dapi, blue. Scale bars = 50 μm. Both male and female 5 - 7 day old adult $SC^{SskRNAi}$ flies demonstrate a significant reduction in viability during (D) (non-dessicating) starvation conditions compared to controls. This sensitivity is absent when these flies are challenged with (E) salt stress, with no significant difference in viability compared with controls. Mantel-Cox (Log rank) test. N’s in parentheses. *P<0.05. **P<0.0001.

Journal of Cell Science • Supplementary information

Figure S7.

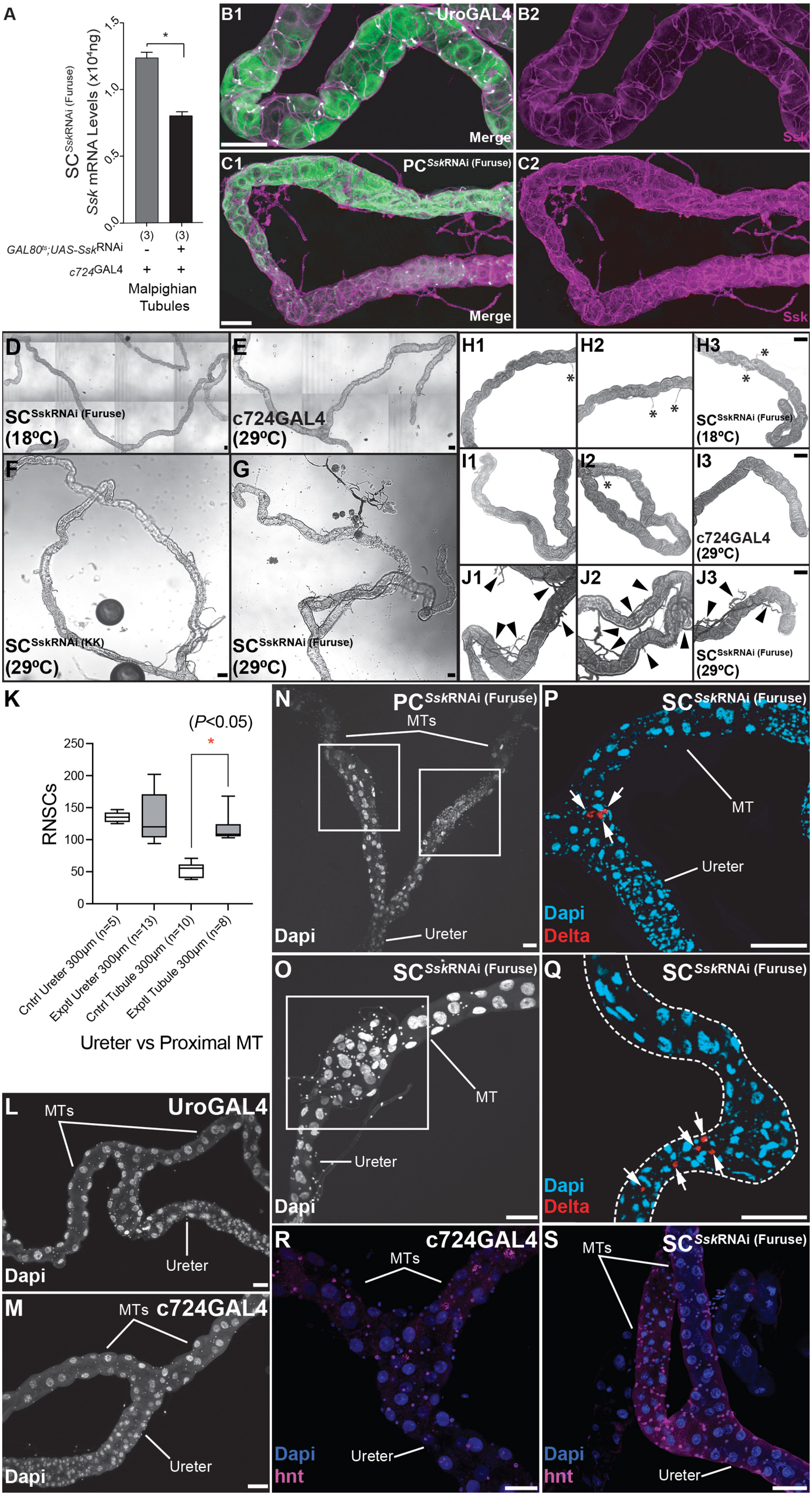

Journal of Cell Science • Supplementary information

Fig. S7. Cell-specific depletion of Ssk results in proliferation of renal nephritic stem cells (RNSCs) and hyperplasia of trachea supplying adult Malpighian tubules.

(A) Quantitative RT-PCR analysis demonstrating ~35% knock down in *Ssk* mRNA expression levels in MTs between 5 day old adult SC$^{Ssk\text{RNAi}}$ flies raised at the restrictive temperature (29ºC) compared with those raised at the permissive temperature (18ºC); 0.802 x 10^{-4} ng ± 0.038 vs 1.237 x 10^{-4} ng ± 0.043 mRNA respectively. *P<0.05, Student's T-Test ±SEM, N's in parentheses. (B) *Uro*GAL4 driving membrane-bound green fluorescent protein (mGFP) in the initial segment day of 5 D adult MT, demonstrating control levels of Ssk expression associated with junctions and, at lower levels, trachea. (C) Hyperplasia of trachea supplying MTs in adult PC$^{Ssk\text{RNAi}}$ flies as realised by anti- Ssk. As with SC$^{Ssk\text{RNAi}}$ flies, hyperplasia levels increase from initial to main segments. Nb- both confocal stacks were collected using identical settings. mGFP, green; Ssk, magenta; Dlg, white. Scale bars = 50 μm. (D) Tiled bright field image of 15 D SC$^{Ssk\text{RNAi (Furuse)}}$ adult MT raised at the permissive temperature (18ºC) demonstrating normal distribution of trachea supplying the MT. (E) Tiled bright field image of 15 D *c724*GAL4>mGFP adult MT raised at the restrictive temperature (29ºC) demonstrating normal distribution of trachea supplying the MT. Bright field images of 15 D SC$^{Ssk\text{RNAi (KK)}}$ (F) and SC$^{Ssk\text{RNAi (Furuse)}}$ (G) adult MTs raised at the restrictive temperature (29ºC) demonstrating hyperplasia of associated trachea supplying the MTs. (H1-3) Details of MTs of 15 D SC$^{Ssk\text{RNAi (Furuse)}}$ adults raised at the permissive temperature (18ºC) demonstrating normal distribution of trachea. (I1-3) Details of MTs of 15 D *c724*GAL4>mGFP adults raised at the restrictive temperature (29ºC) demonstrating normal distribution of trachea. (J1-3) Details of MTs of 15 D SC$^{Ssk\text{RNAi (Furuse)}}$ adults raised at the restrictive temperature (29ºC)

Journal of Cell Science • Supplementary information

demonstrating hyperplasia of associated trachea. Asterisks indicate normal distribution of trachea. Arrowheads indicate hyperplastic trachea. Scale bars = 50 μm. (K) Graphical representation of comparative 'tiny' renal nephritic stem cell (RNSC) counts between c724GAL4 control and SC$^{Ssk\text{RNAi}}$ experimental adult MTs, made along a 300μm length of the ureter (starting from the top of where the ureter bifurcates into the tubules), and a 300μm length along the proximal segment of the tubules (from where it joins in to the ureter). Demonstrating a significant increase in the RNSC population in the proximal segment of the tubule, but no difference in the RNSC cell population associated with the ureter. Box-plot whiskers, min to max. *P<0.05, Student's T-Test, N's in parentheses. 5 day UroGAL4 (L) and c724GAL4 (M) control adult MTs exhibiting normal distribution of RNSCs nuclei in the ureter and proximal segment of the MTs. 5 day PC$^{Ssk\text{RNAi}}$ (N) and SC$^{Ssk\text{RNAi}}$ (O) experimental adult MTs exhibiting 'bulbar' areas with proliferation of RNSCs' nuclei (white boxes) in proximal segment. Dapi, white. Scale bars = 50 μm. 5 day SC$^{Ssk\text{RNAi}}$ adult MTs ureter, proximal (P) and main segment (Q) exhibiting co-expression of the proliferative cell marker anti-Delta (Delta) with RNSCs' nuclei (arrows). Dapi, blue; Delta, red. 5 day c724GAL4 (R) control and SC$^{Ssk\text{RNAi}}$ (S) experimental adult MTs exhibiting co-incident expression of the proliferative cell marker anti- hindsight (hnt) with RNSCs' nuclei (arrows) in the ureter and proximal segment. Dapi, blue; hnt, magenta. Scale bars = 50 μm.

Journal of Cell Science • Supplementary information

Figure S8.

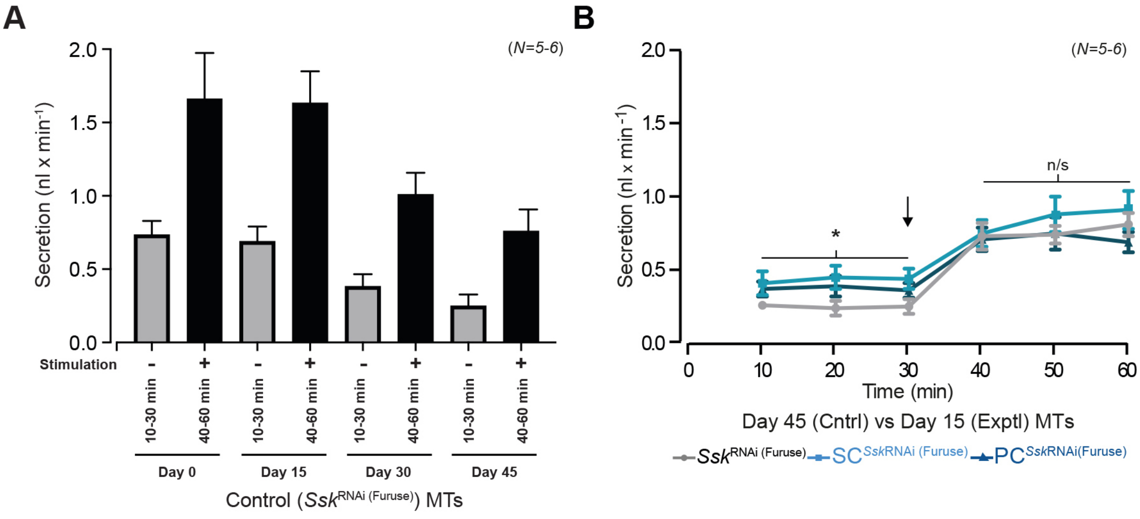

C

	Control (*Ssk*RNAi) (nl x min-1)		SC*Ssk*RNAi (nl x min-1)		PC*Ssk*RNAi (nl x min-1)	
Day/Time	0-30 min	40-60 min	0-30 min	40-60 min	0-30 min	40-60 min
Day 0	0.733 ±0.019 *N*=6	1.663 ±0.033 *N*=6	0.673 ±0.014 *N*=6	1.463 ±0.043 *N*=6	0.630 ±0.006 *N*=6	1.380 ±0.032 *N*=6
Day 15	0.690 ±0.012 *N*=3	1.563 ±0.012 *N*=3	0.373 ±0.009 *N*=6	0.717 ±0.018 *N*=6	0.433 ±0.012 *N*=4	0.847 ±0.049 *N*=4
Day 30	0.383 ±0.017 *N*=6	1.010 ±0.035 *N*=6	0.170 ±0.015 *N*=6	0.573 ±0.023 *N*=6	0.150 ±0.100 *N*=6	0.513 ±0.035 *N*=6
Day 45	0.250 ±0.006 *N*=3	0.760 ±0.025 *N*=3	0.097 ±0.012 *N*=5	0.473 ±0.014 *N*=5	0.133 ±0.009 *N*=4	0.510 ±0.035 *N*=4

Fig. S8. Cell-specific *Ssk* depletion accelerates the age-related decline in function in adult Malpighian tubules.

(A) Graph of basal (grey) and stimulated (black) secretion rates in control adult MTs over progressive time points, demonstrating the natural decline in tubule functional capacity that occurs as flies age. (B) Comparison of fluid secretion rates between 45 day old control and 15 day old PC^{SskRNAi} and SC^{SskRNAi} flies experimental adult MTs. While experimental MTs basal secretion rates are marginally better, there is no significant difference in secretion rates upon stimulation with DromeKinin (black arrow). *P<0.05, paired samples t-test ±SEM, N's in parentheses. (C) Table of fluid secretion rates (nl x min^{-1}) ± SEM for time points 0-30 min (unstimulated) and 40-60 min (stimulated with 10^{-6} DromeKinin) for control and experimental (SC^{SskRNAi} and PC^{SskRNAi}) adult tubules from day 0 to day 45. N's indicated.

Journal of Cell Science • Supplementary information
